# Metabolism at Evolutionary Optimal States

**DOI:** 10.3390/metabo5020311

**Published:** 2015-06-02

**Authors:** Iraes Rabbers, Johan H. van Heerden, Niclas Nordholt, Herwig Bachmann, Bas Teusink, Frank J. Bruggeman

**Affiliations:** 1 Department of Systems Bioinformatics, VU University Amsterdam, De Boelelaan 1085, 1081 HV Amsterdam, The Netherlands; E-Mails: j.van.heerden@vu.nl (J.H.H.); n.nordholt@vu.nl (N.N.); h.bachmann@vu.nl (H.B.); b.teusink@vu.nl (B.T.); f.j.bruggeman@vu.nl (F.J.B.); 2 NIZO Food Research, 6718 ZB Ede, The Netherlands; 3 Top Institute Food and Nutrition, 6700 AN Wageningen, The Netherlands

**Keywords:** evolution, metabolism, optimality, fitness landscapes, selection pressure, trade-offs

## Abstract

Metabolism is generally required for cellular maintenance and for the generation of offspring under conditions that support growth. The rates, yields (efficiencies), adaptation time and robustness of metabolism are therefore key determinants of cellular fitness. For biotechnological applications and our understanding of the evolution of metabolism, it is necessary to figure out how the functional system properties of metabolism can be optimized, via adjustments of the kinetics and expression of enzymes, and by rewiring metabolism. The trade-offs that can occur during such optimizations then indicate fundamental limits to evolutionary innovations and bioengineering. In this paper, we review several theoretical and experimental findings about mechanisms for metabolic optimization.

## 1. Environments, Natural Selection and Fitness

**Figure 1:**
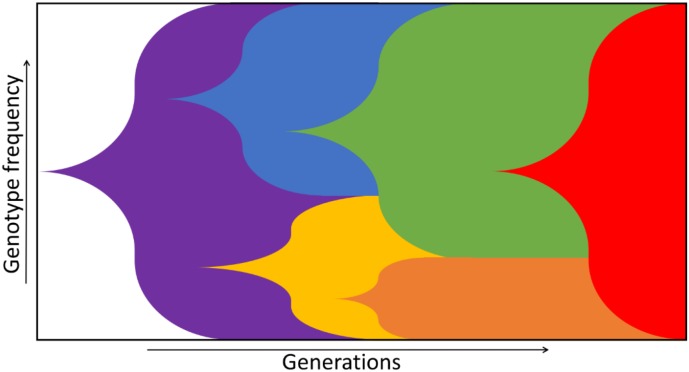
A Muller plot depicting the succession of different genotypes (colours). New genotypes arise that can fill certain niches (blue, yellow) or take over the whole population by outcompeting others (red). This leads to a new composition of the population in a specific environment. Adapted from Barrick *et al.* [1].

Natural selection is the process by which the relative frequencies of genotypes in a population change. In microbial populations, new genotypes typically arise through mutations. Natural selection can act upon mutations when they lead to a change in phenotype, which, in turn, can lead to an increase or decrease in the frequency of the associated genotype. In time, the genetic makeup of a population is characterised by the emergence, co-existence and succession of different genotypes (Figure 1) [1]. Which genotypes increase in frequency depends on their fitness in the prevailing environment, relative to the fitnesses of the current competing genotypes, setting the selection pressure.

Fitness is determined by many variables and is dependent on environmental conditions. High fitness at constant conditions of nutrient excess requires, for instance, different traits than during constant nutrient limitation. Consider two genotypes, one with a high maximal growth rate and low nutrient affinity, and another with low maximal growth rate and high nutrient affinity. A critical nutrient concentration exists for this example, above which the fast grower wins whereas below it the slow grower wins. The same applies during dynamic conditions: the genotypes that are good at dealing with irregular switches between nutrient excess and absence may not be successful in dealing with antibiotic challenges as they might lack the necessary resistance genes.

What other factors determine the phenotypic traits that characterize a fit genotype? In addition to the environmental conditions, fitness depends on cellular and evolutionary constraints. Cellular constraints are constraints inherent to the molecular composition of a cell. They are imposed by limitations related to i.a. biophysics (e.g., limitations to diffusion rate) and biochemistry (e.g., overexpression of proteins can lead to a situation where benefits no longer outweigh the costs). Evolutionary constraints on the other hand relate to limitations in the movement over a fitness landscape, as a result of i.a. epistatic effects and the existence of adaptive valleys (see Section 2).

An example of cellular constraints would, for instance, occur when a population of bacteria is suddenly exposed to a high dose of a potent antibiotic. Some genotypes may run the risk of dying if they fail to adapt their phenotype fast enough, say via the induction of an antibiotic-degrading enzyme. An adaptive strategy that relies solely on antibiotic sensing and subsequent gene expression of the required genes will then often be ineffective, as irreparable cell damage can occur before enough antibiotic-degrading enzymes are synthesized; so, those genotypes are too slow. One solution is that genotypes evolve anticipation capacities for antibiotic additions. Cells could, for instance, always express antibiotic-degrading enzymes or, alternatively, diversify a population into one with different phenotypes, such that a subpopulation of cells is always prepared for the sudden presence of antibiotics. A population diversification strategy could be beneficial if synthesis of antibiotic-degrading enzymes is a too high fitness burden in the absence of the antibiotic. The response time of a genotype therefore puts a constraint on the repertoire of feasible adaptive strategies. This example also indicates that genotypes with different strategies can in principle compete: one genotype could be anticipatory while another diversifies its population.

Figuring out how microbial evolution works requires understanding of the phenotypic traits that contribute to fitness in different environments. In some environments, genotypes with high growth rates may be selected whereas in others high cell yield or stress resistance pays off. Appreciation of cellular constraints matters as well, since they impose limits to feasible adaptation strategies. Cells have, for instance, limited resources and proper management of their protein economy is therefore another cellular constraint for fitness traits [2,3,4,5,6].

**Figure 2:**
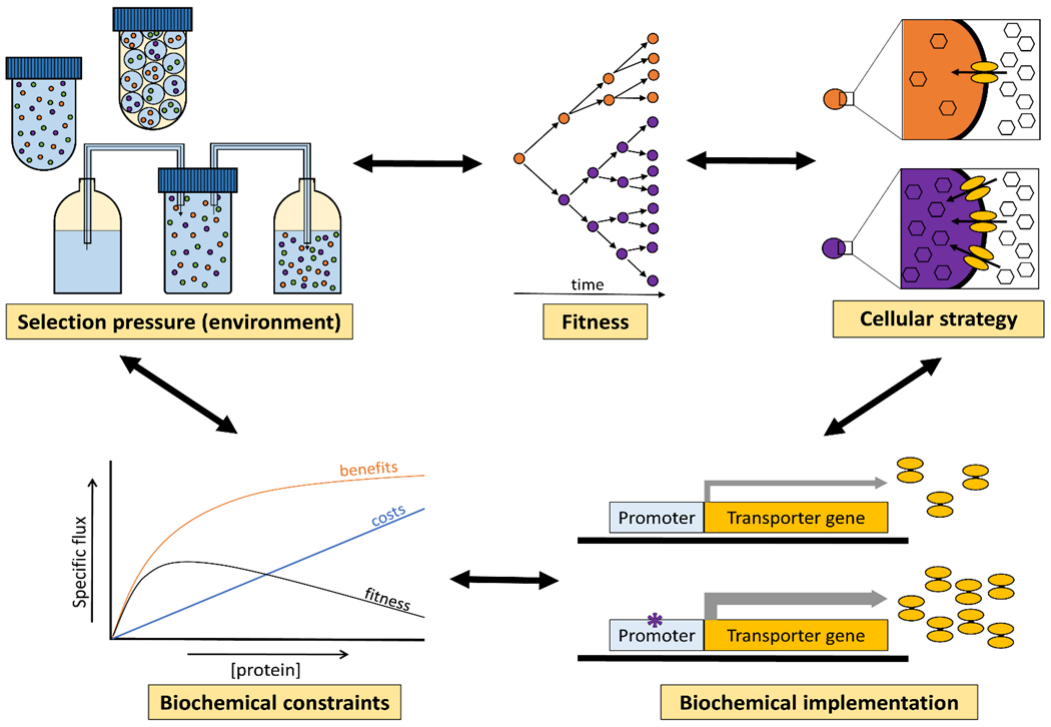
In evolution, the selection pressure that an organism is exposed to is set by its environment (e.g., culturing method; available substrates; presence of competitors). Fitness is defined as the ability to reproduce and/or survive in a certain environment. Different cellular strategies might increase the fitness of an organism, for example by increasing the substrate uptake rate. Cellular strategies have a biochemical basis. Adaptive changes in the biochemistry of a cell, due to a mutation, are constrained. Too high expression levels, for instance, eventually lead to a decrease in fitness when the benefits of protein production no longer outweigh its costs. There is interaction in both directions between these levels, as on the one hand a change in the environment can be a driving force for cellular adaptation through mutation and selection, but on the other hand a change in cellular strategy might change the environment (e.g., by producing metabolic by-product like acetate).

Understanding which molecular properties of a cell enhance fitness is therefore a major challenge, and requires: (i) understanding of the impact of mutations on protein properties, e.g., their kinetics; (ii) understanding the role of proteins in the biochemical reaction network of the cell; (iii) relating fitness to the systemic properties of the underlying biochemical network; and (iv) identification of proteins that determine those systemic properties. In our view, addressing these problems requires merging of theories of biochemistry, systems biology and evolutionary biology (see Figure 2).

In this review, we explore several successful examples of the integration of biochemistry, systems biology, and evolutionary theory. We will limit ourselves mostly to the evolutionary aspects of metabolism and cell growth.

## 2. Fitness Landscapes

**Figure 3:**
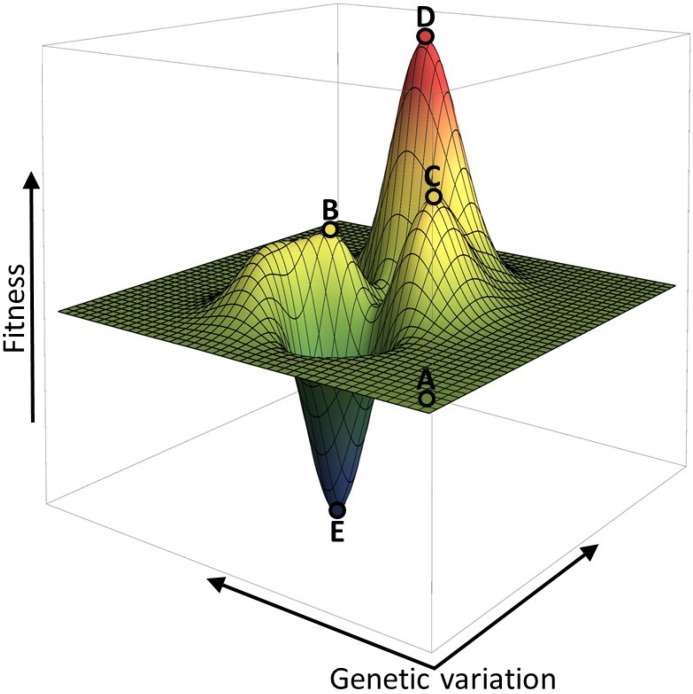
The fitness of an organism is often visualized as an adaptive landscape, with genomic changes (*i.e.*, point mutations in genes or regulators) on the x- and y-axes, and fitness on the z-axis. Most mutations are expected to have a neutral (point A) or negative (point E) effect on fitness. Some mutations however can lead to fitness increases (B, C, D). The genetic background of an organism (the location on the landscape), can have a deterministic effect on the adaptive path. For example, it is virtually impossible for an organism at point C to evolve to point D, as it would have to overcome an “adaptive valley”, *i.e.*, acquire mutations with a negative effect on fitness before it can increase its fitness again.

The view of evolutionary adaptation as a walk over a rugged fitness landscape was already introduced in 1932 by S. Wright [7]. Such a landscape simplifies the fitness-genotype relation to one that can be visualised in a 3D-plane. In this plane, each (X,Y) coordinate represents a unique DNA sequence or cellular state in a given environment. Genetic alterations (for example, point mutations in genes, or amino acid substitutions in proteins) can be portrayed as motion over this plane. Each sequence has a fitness value, depicted as the Z-value. Figure 3 illustrates this concept and indicates also that genetic variation can either have a neutral effect (the flat plane around point A), a positive effect (points B, C and D) or a negative effect (point E) on fitness. 

In this framework, evolutionary adaptations can thus be seen as a stepwise change of the genotype—along the X, Y-axes—accompanied by alterations in fitness (the Z-value). Mutations might for example enhance stress resistance of a cell, or enable it to outgrow competitors. Genetic changes can be mediated by sequential mutations that each lead to a gradual fitness increment (small slope towards point B in Figure 3). Alternatively, only a few mutations induce a huge fitness increase (steep slope towards point D in Figure 3).

Mutations arise randomly. Out of all possible sequence changes, the majority is expected to have no effect on fitness (50%–70%), a large portion is likely detrimental to fitness or even leads to a complete loss of viability (30%–50%), while only very few mutations are expected to be beneficial (0.01%–1%) [8]. As a result of positive selection, a mutation that is neutral or beneficial may be maintained in the population or even take it over and become fixed. On the other hand, a mutation that, for instance, renders a protein less functional will typically give rise to an organism that is outcompeted by its ancestor and this mutation will be lost from the population [1]. Generally speaking, only functional mutants of a protein can be maintained in the population [9]. Protein evolution through random mutation and selection therefore often consists of gene duplication before mutations are accumulated [10]. An adaptive walk over a fitness landscape will accordingly only move over a flat surface (neutral mutations) or move uphill (beneficial mutations), while downhill movement (detrimental mutations) is considered unfavourable—as the corresponding genotypes reduce in frequency in the population. In the example in Figure 3, organisms in the genetic state of peak C are thus not expected to reach the peak D; they are stuck at an “evolutionary trap”. Reaching D would first require mutations that decrease fitness before fitness can increase as a result of a second set of mutations (often referred to as an adaptive valley). We note that some mutational processes, such as recombination in eukaryotes [11] and horizontal gene transfer in bacteria [12] can allow for such large jumps in the fitness landscape, passing over local fitness wells.

Studies on epistasis show that the evolutionary history is one of the main determinants for the available adaptive trajectories over a fitness landscape [1,8,13,14,15,16]. Epistasis is a widely occurring phenomenon that describes how the fitness effects of one mutation might depend on other mutations (*i.e.*, the “genetic background”). When an epistatic interaction between mutations is positive (synergistic epistasis), one of the mutations has a higher fitness gain if the other “potentiating” mutation already exists. Two types of negative epistatic interactions can be distinguished: (i) “diminishing returns” or “antagonistic epistasis” and (ii) “sign epistasis”. In the case of diminishing returns, the fitness effect of combined mutations is less than the sum of all individual mutations. In the case of sign epistasis, the effect of the combined mutations is the opposite of the effect of the individual mutations. Fitness enhancing mutations then become fitness reducing when occurring together.

Diminishing returns epistasis is thought to occur most frequently. This phenomenon is illustrated by Chou *et al.* [17]. They replaced a native pathway that is essential for methanol growth in *Methylobacterium extorquens* by a foreign pathway from *Paracoccus denitrificans*. This led to a severe reduction in growth rate. They described four specific mutations that arose in an evolution experiment on methanol. Each mutation partially restored the growth rate. All 16 possible combinations of these mutations were subsequently constructed. They discovered that the fitness advantages of these mutations, rather than being additive, gave rise to reduced selective advantages when they were introduced into a more fit genotype that already included one or several fitness enhancing mutations. The authors explained these results by a benefit-cost optimisation model, where rather than conveying a direct effect on fitness, the mutations affected the costs and benefits of protein production.

The environment of an organism is one of the factors that shapes the genotype-fitness landscape. The landscape in Figure 3 might have a very different topology when the organism is subjected to a new environment, where the number, heights and positions of fitness peaks and valleys are different. This effect was illustrated by de Vos *et al.* [18]. They used *Escherichia coli LacI* mutants that upon three particular mutations inverted their response to the addition of IPTG—gene repression as opposed to induction. Those mutations therefore changed the fitness effects of IPTG addition in an environment with glucose from negative (costly production of unneeded protein) to positive (reduction of protein expression). They tested all possible trajectories of mutation combinations in the presence and absence of IPTG and concluded that the observed epistatic effects were strongly dependent on the environment. For instance, under one condition a certain mutation trajectory can be inaccessible while it can be under positive selection under another condition. De Vos *et al.* [18] therefore illustrated that the feasible trajectories over a fitness landscape are environment dependent. This indicates that environmental factors can prime a genotype for certain genetic adaptations.

The speed of movement over a fitness landscape is greatly influenced by the mutation rate. This rate influences both the evolvability and plasticity of an organism as well as the genetic robustness of its phenotype. Plasticity refers to the ability of an organism to generate offspring with variable traits in order to adapt to its environment. Genetic robustness refers to the ability to fix beneficial traits and avoid detrimental or deleterious mutations [1,15,16,19,20]. When organisms are faced with a new environment, or variable conditions, the selection pressure on adaptive mutations will be high and the enhanced mutation rate appears advantageous. It has indeed been shown several times, especially during the first part of evolution experiments, that the mutation rate of populations was increased. One example comes from a long-term evolution experiment (LTEE), where, out of 12 replicate populations of *E. coli*, six evolved hyper mutability, with a point mutation rate up to 150-fold higher than that of the ancestor [21,22]. Hypermutators could arise via a mutation in the *mutT* gene, involved in mismatch repair. The mutator phenotypes had a selective advantage. Their rates of fitness increase and the final fitness reached were higher than that of the strains with the ancestral mutation rate, over the course of 50,000 generations [22]. Strains that are adapted to a constant environment can experience negative effects of hyper mutability, as it also increases the chance of detrimental mutations (often referred to as “genetic load”). This was confirmed by the finding that, in the later stages of the LTEE, a mutation in *mutY* occurred that reduced the mutation rate by 40%–60% [21]. In other experiments, like those of McDonald *et al.* [23], in which yeast hypermutators evolved under constant conditions, a severe reduction in mutation rate occurred as well. In conclusion, changing environments can favour a high mutation rate to achieve high evolvability, while adaptation to constant conditions can favour reductions in mutation rate to allow for high robustness.

The study by Weinrich *et al.* [24] provides a good example of how protein evolution can be experimentally linked to fitness. They looked at the evolution of cetofaxime resistance of *E. coli*, describing it as an adaptive walk over a fitness landscape. It is known that a ~100,000 fold increase in antibiotic resistance can result from five specific point mutations in β-lactamase. A total of 32 possible combinations were constructed in *E. coli*. This strain library revealed a single-peaked fitness landscape, with no suboptimal intermediates that showed reduced fitness and there were no adaptive valleys. The highest fitness occurred when all five mutations were present. A total 120 possible trajectories for those five mutations (representing the different orders in which these mutations could have been acquired) were all constructed. Out of them, 102 trajectories were found to be evolutionary inaccessible, due to landscape ruggedness or sign epistasis. For most of the remaining 18 trajectories the order of mutation occurrence was essential, and only few trajectories were very likely. The protein biochemistry, underlying the effects of the mutations, offers some insight. There appears to be an interplay between the hydrolytic, aggregative and thermostability properties of the β-lactamase upon mutations (referred to as pleiotropy; one mutation gives rise to several effects at once). Since many mutational trajectories were infeasible, and some much more likely than others, the authors conclude that “replaying the protein tape of life might be surprisingly repetitive”. Similarly, the replacement of an adenylate kinase (*adk*) in the thermophilic bacterium *Geobacillus stearothermophilus* with the *adk* gene from the mesophilic bacterium *Bacillus subtilis*, and the subsequent adaptation in turbidostat to high temperature, showed the same 5 mutations occurring in *adk* in hundreds of isolated mutants [25]. It was further shown that these 5 mutations impacted on protein folding and activity, which was critical for this evolutionary outcome [26]. This phenomenon is further supported by studies that compare empirical adaptive walks to simulated ones, where fitness is randomly assigned to a genotype. Experimental adaptive walks appear as non-random, in contrast to the emergence of the required mutations. The number of available adaptive mutations and the magnitude of the selective pressure does however play a role [27,28].

We conclude that evolutionary trajectories are shaped by limitations imposed by selective pressures, epistasis, the environment and robustness. Together, these factors have severe implications for the number of feasible paths over a fitness landscape.

## 3. Selection Pressures Imposed by the Environment

The environment of an organism mainly defines the selection pressure. It is therefore important to gain an understanding of how selection pressure varies across environmental conditions. In this section we will discuss a number of different environments, the selection pressures that they impose and how fitness maximising strategies rely on aspects of the biochemistry of metabolism.

### 3.1. Experimental Culturing Methods

**Figure 4:**
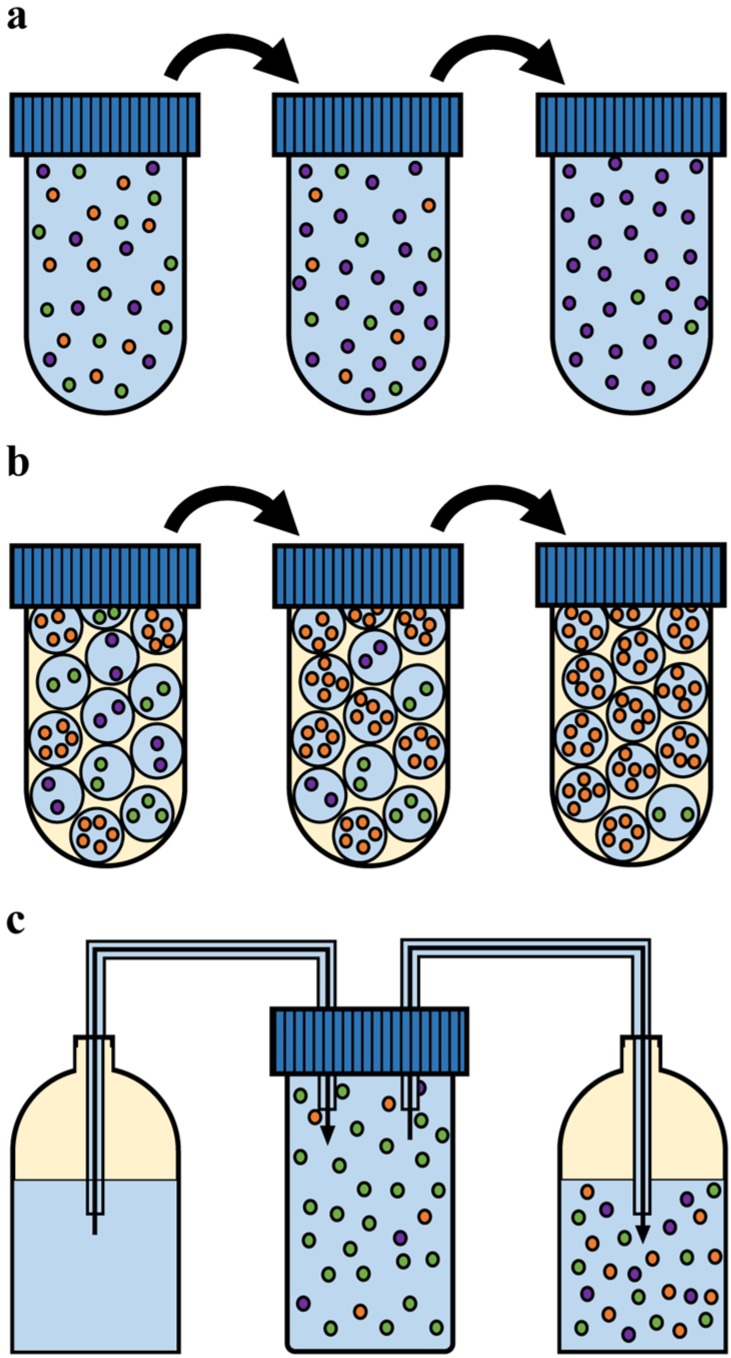
Culturing methods used in laboratory evolution experiments. (**a**) Serial propagation in batch, where after a certain period of growth in suspension, cells are diluted into fresh medium and the cycle is repeated; (**b**) Serial propagation in emulsion, where single cells are compartmentalized in a water-in-oil emulsion, thereby privatizing the resources. Each cell is now allowed to deplete the resources before the cells are mixed, diluted and a new emulsion is made. This cycle is subsequently repeated; (**c**) During chemostat culturing, a continuous inflow and outflow of medium ensure constant growth rate and conditions for the cell population. In all figures the different colours indicate different genotypes.

Microbial populations can be subjected to numerous different environments. The best characterized environments are those that can be imposed in the laboratory: culturing in batch, in chemostat and, recently, also in emulsion (see Figure 4 and Table 1). Each culturing method imposes a different selective pressure and, in principle, allows for the selection of mutations that are beneficial in one environment and detrimental in another. For instance, organisms with a high maximal growth rate on glucose in batch do not necessarily outcompete slow glucose-growers in a chemostat run at low dilution rates.

Besides changing the culturing method, conditions can be changed. For example, the medium can be varied from rich to minimal medium and different nutrients and stresses, including antibiotics, can be considered.

**Table 1:** Summary of main properties of experimental culturing methods.

	Batch	Emulsion [29]	Chemostat
**Fitness measure**	Growth rate (μ)	Number of cells (N)	Substrate concentration [S] at dilution rate (D) = μ
**Evolutionary objective**	Maximize μ	Maximize N	Minimize [S] at D = μ
**Possible strategies**	High μ_max_	(a) High metabolic yield (b) Small cells	(a) Low K_M_ (b) High μ_max_
**Examples**	Fermentation in yeast; overflow metabolism in *E. coli*	Mixed acid fermentation in *L. lactis*	Metabolic switches
**Trade-offs**	Low yield	Low μ_max_	(a) Low μ_max_ (b) Low yield
**Method for prediction of optimality**	EFM + enzyme kinetics	FBA	EFM + enzyme kinetics

By changing the culturing method and the growth conditions, such as medium and stresses, one can vary factors contributing to fitness. Performing an experiment with similar conditions, but different culturing methods, may lead to a different evolutionary outcome. By making specific mutants compete against each other, or by starting with an isogenic population and allowing evolution to run its course, genotypes that prove fit in a certain environment can be identified.

In this section, we discuss several evolutionary implications for microorganisms that are subjected to different culturing methods. We will discuss a number of examples of adaptive strategies that increase fitness. Two culturing methods we will not discuss in great detail are the turbidostat [30] and the auxostat [31] which are similar in setup to a chemostat. For a turbidostat, the dilution rate is set by the turbidity of the culture in order to keep it constant. The turbidostat therefore allows for the evolutionary maximization of the maximal growth rate of microorganisms at constant conditions. The auxostat does not keep the turbidity fixed but another growth-related variable, such as the pH or CO_2_-level [31]. Two studies where the turbidostat was employed [25,68] are discussed in Section 2 and Section 3.2.

#### 3.1.1. Batch Cultivation

Serial dilution in batch is a method that has been employed in biological research for many decades. A population of microbes is grown in a suspension culture, after which (depending on the research objective during the exponential growth phase or in stationary phase) a fraction of this population is propagated into fresh medium and the cycle is repeated (see Figure 4a). Batch culturing is characterized by direct competition of microbes for the available resources during growth, followed by a bottleneck, the dilution step, when a random sampling of the grown population occurs.

Many evolutionary experiments have been conducted using serial dilution in batch. The most well-known study was started in 1988 by Lenski, using 12 replicate cultures of *E. coli* [22]. It has been running for over 50,000 generations now. An important finding was that the growth rate (fitness) of all replicate populations increased rapidly during the first few thousand generations, after which the rate gradually declined (diminishing returns). They found that a hyperbolic model, where the growth rate goes towards a maximum, underestimated the growth rate in the later generations. A power-law model described the long-term trends of fitness increase over time much better. In such a model, no upper bound is formulated, and the used parameters are mutation rate, beneficial effects and diminishing returns. Their conclusion was that evolution can keep increasing fitness (growth rate) for billions of generations in a constant environment, albeit at ever lower rates of increase.

Ibarra *et al.* [32] also undertook an evolution experiment in batch by serial dilution. They used a stoichiometric model of *E. coli*’s metabolism to predict optimal values of oxygen and substrate uptake rates that maximize growth rate for various carbon sources. These predictions were validated by evolution experiments. *E. coli* was cultured in batch for 500–1000 generations on malate, acetate, glucose and succinate, leading to growth rate increases ranging between 17%–21%. The evolved uptake rates and growth rates of the wildtype and evolved strains were in agreement with the model. Glycerol turned out to be a special case. Only after prolonged evolution, measured rates moved towards optimal values and growth rates increased over 50% for the evolved strains.

While the last two examples were based on the prokaryote *E. coli*, the phenomenon that cultivation in batch leads to enrichment of a high growth rate phenotype has also been shown for other microorganisms, like the eukaryote baker’s yeast, *Saccharomyces cerevisiae* [33,34]. The authors found growth rate increases of up to 80% in the first 2000 generations of evolution, while the subsequent 3000 generations led to only a small increase in growth rate of a few percent (in line with the diminishing returns observations of [22]). The total increase in growth rate was dependent on the population size, with higher increases for larger populations, as the large population size entailed a larger genetic variance. Growth rate increases correlated negatively with growth yield (see Section 4.3).

Many examples therefore exist of microorganisms that display increased growth rates after prolonged cultivation in batch. However, what the underlying adaptive mechanisms are, in terms of the biochemistry of metabolism, often remains unclear. Mathematical analysis of metabolism models has recently solved one part of this puzzle. Two recent papers, by Wortel *et al.* [35] and Müller *et al.* [36], show that the optimal state of metabolism that maximises the cellular growth rate, or more generally any reaction rate normalised by the total cellular protein investment, is necessarily an elementary flux mode (EFM). An EFM is a metabolic network that operates at a thermodynamically feasible steady state and from which no reaction can be removed without violating the steady state constraint [37]. Since an EFM is in essence a single metabolic route, this finding indicates that growth rate is increased by eliminating alternative routes in metabolism. The statement that an EFM is the optimal metabolic state is independent of the kinetic parameters of all the metabolic enzymes, which makes this prediction quite remarkable. Which EFM, out of all alternative EFMs, is the optimal one however does depend on the precise enzyme kinetics and the imposed environmental conditions (nutrient concentrations).

In conclusion, culturing in batch puts substantial selection pressure on the growth rates of microorganisms [38]. Increased growth rates are often the result of rewiring of the underlying metabolic networks. In the evolutionarily optimal state, theory predicts that the active metabolic network is an EFM.

#### 3.1.2. Emulsion Cultivation

In 2007, Schuster *et al.* [39] compared the maximisation of metabolic fluxes and yields. A metabolic yield is defined for any flux route and gives the stoichiometry of the net conversion. For instance, the growth rate divided by the uptake rate of a substrate corresponds to the cell yield on that substrate, given the gram biomass that can be obtained from a mole substrate. Using a simplified model, they showed that rate and yield maximizations are not equivalent. Another theoretical method, flux balance analysis (FBA), popular in biotechnology, predicts flux distributions through metabolism that optimize the yield of a product of metabolism, such as biomass, a biofuel or ATP. It should be noted that in the past, authors have reported the use of FBA to predict optimal strategies for growth rate maximisation, however, this is not strictly correct [39,40]. When FBA results were compared to experimental results, it was often found that flux-distribution predictions were not observed. For example, a fast growing yeast relies on a low-yield mode of metabolism. It partially ferments glucose into ethanol, with relatively low ATP yield per glucose, rather than respiring all glucose into CO_2_. It is likely that experimental data from batch cultivations are in better agreement with the optimisation of metabolic rate than yield [39]. Yield selection is perhaps also not so common in experimental evolution experiments. This has recently changed through the introduction of emulsion cultivation methods [29].

It has often been suggested that a trade-off exists between biomass yield and the growth rate of microorganisms [32,33,39,41,42,43,44] (see Section 4.3). Accordingly, evolution in batch, which selects for growth rate (metabolic flux), is expected be accompanied by a reduction of the biomass yield. Pfeiffer *et al.* [45] proposed, some time ago, that a spatially-structured environment would eliminate competition between cells, and shift the selection pressure on growth rate to biomass yield. Such a novel method has recently been introduced by Bachmann *et al.* [29]. Individual cells are compartmentalized in a water-in-oil emulsion, grown to stationary phase, subsequently mixed together and diluted into fresh medium after which to cycle is repeated (see Figure 4b). In this method, nutrients (resources) are privatized per genotype, while a large enough population for evolution to act upon is maintained. Serial propagation in emulsion selects for variants with the highest number of viable offspring. This can be achieved for example by producing smaller, but more, cells or by increasing the metabolic yield. As there is a lower limit to cell size, this method will eventually select for increased metabolic yield, and hence the term “yield selection” is regularly used.

Bachmann *et al.* [29] showed that prolonged culturing in emulsion leads to selection of high yield *Lactococcus lactis* genotypes [29], reflected in the observed increase in final OD, cell number, total protein content and dry weight. These yield increases coincided with reduced growth rates. The high yield mutant had switched from lactic-acid metabolism (high flux, but a low yield of ~2 ATP/glucose) to mixed acid metabolism (low flux, increased yield of ~3 ATP/glucose). In addition, the cell volume had decreased. These two phenotypic changes can account for the increased cell number and biomass yield. Sequencing revealed a point mutation in the main glucose transport system, that led to reduced glucose transport and glycolytic flux, which caused the observed metabolic switch. This mutation therefore explains the high yield/low rate phenotype. Subsequent culturing in batch of the evolved high-yield mutant caused the emergence of revertant phenotypes that had increased growth rates and a reduced yield; they had switched back to homolactic metabolism. This experiment demonstrates the strong selective pressure against high yield/low rate mutants when nutrients are not privatized and substrate competition occurs.

#### 3.1.3. Chemostat Cultivation

Chemostat cultivation (Figure 4c) is a method that allows for steady state growth in a constant environment. A population of cells is maintained in a reaction vessel where fresh medium is continuously supplied and an outflow ensures that the culture volume stays constant. Cells can only maintain themselves in the vessel if they grow equally fast as the dilution rate of the medium. In addition, a stirrer keeps the culture well suspended and the culture pH is controlled.

The selection pressure changes with the dilution rate (D) of the chemostat. The microorganisms (*i.e.*, genotypes) that win the competition in the chemostat are those that can attain the growth rate—the dilution rate, set by the experimentalist—at the lowest concentration of the limiting nutrient. This is regardless of the value of the dilution rate, *i.e.*, whether it is far below or close to the maximal growth rates of the microorganisms. Which cellular growth parameter is selected for however depends on the growth rate.

**Figure 5:**
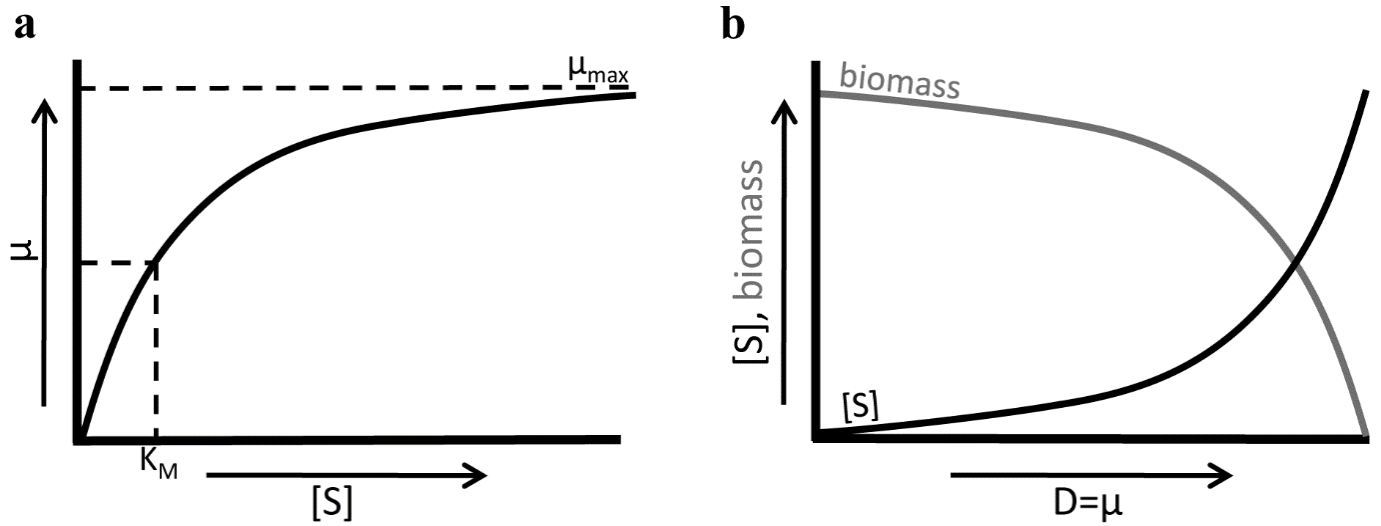
Jacques Monod’s relation of bacterial growth (**a**) in which the growth rate (μ) goes towards an upper limit (μ_max_) at high substrate concentrations ([S]) and the substrate affinity is depicted as the Monod constant (K_M_), the substrate concentration at half the maximal growth rate. The Monod equation has implications for culturing in chemostat; (**b**) the growth rate is set by the dilution rate in the chemostat. The substrate concentration increases with the dilution rate (D) of the chemostat, again according to the Monod relation, thereby changing the selection pressures on microbes cultured at different Ds. At low growth rate the Monod constant is likely more important as a selection target whereas at high growth rates the maximal growth rate becomes more important, as is the case in batch cultures.

**Figure 6:**
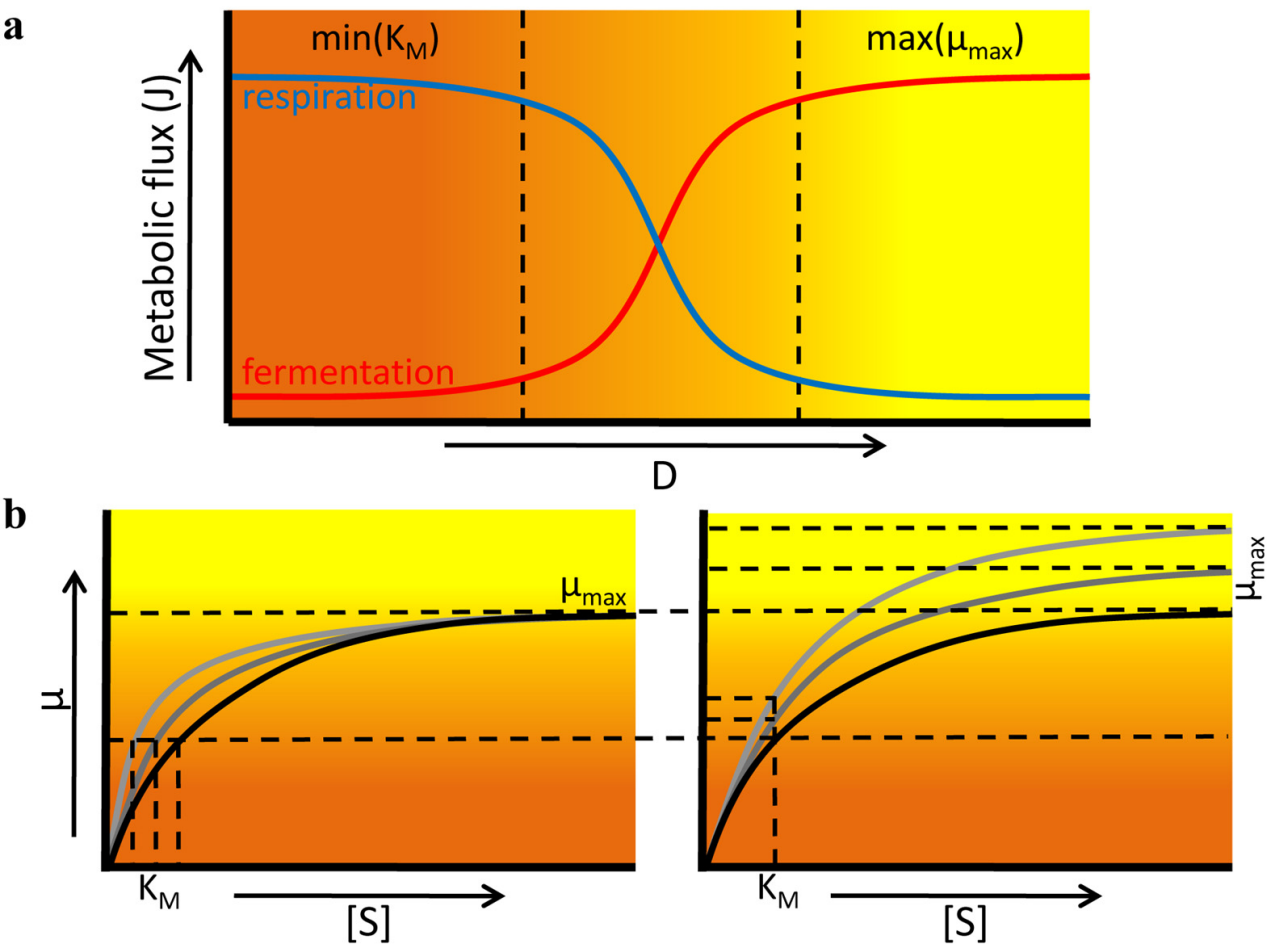
Different dilution (D) rates in a chemostat lead to different selection pressures. (**a**) While the metabolic rate increases at increasing D, for example through increasing utilization of the fermentative strategy, the metabolic yield will decrease at higher D as the flux through the respiratory pathway goes down [43]; (**b**) At low dilution rates, it is more advantageous to increase substrate affinity, and low K_M_ will be selected for. At higher dilution rates on the other hand, increases in μ_max_ will give the bigger selective advantage.

The Monod relationship (Figure 5) relates the growth rate (*μ*) of a microorganism to the concentration *s* of the growth limiting nutrient in the chemostat. The Monod relation, $\mu =\mu_{max}\frac{s}{s+K_{M}}$, depends on two constants: the Monod constant (*K_M_*, but often also referred to as *K_S_*) and the maximal growth rate (*μ_max_*). At steady state, the growth rate equals the dilution rate (D). If a new genotype (mutant) appears, then it outcompetes the resident genotype when it can attain the dilution rate as its growth rate, at a lower concentration of *s*. When the mutant appears, the concentration of *s* is set by the resident. Then, the mutant grows faster than the resident, as it grows faster at a lower *s*. The mutant now becomes more abundant. It starts to determine the concentration of the limiting substrate in the reactor more and more, thereby slowly reducing this concentration. While this is happening, the resident starts to grow at a rate lower than the dilution rate, and flows out. The mutant that won either has a changed *K_M_*, *μ_max_*, or both. At high *s*, when *s* is much greater than *K_M_*, the growth rate is close to the maximal growth rate. Any change in the value of *K_M_* now has a negligible effect (Figure 6b). When the dilution rate is reduced and the organism is no longer growing at a close to maximal growth rate, the *K_M_* starts to become increasingly important. So, at low to intermediate dilution rates mutations affecting *K_M_* and $\mu_{max}$ can be fixed, whereas at a high dilution rate the $\mu_{max}$ is selected for. Hence, the selection pressure can be on different growth parameters in the chemostat. This was not the case in the batch and emulsion based cultivation method.

The selection for increased substrate affinity—a lower K_M_—in chemostat has been observed many times. Jansen *et al.* [46], for example, observed a large decrease in the K_M_ and only a slight increase in the V_max_ of the glucose transporter, upon prolonged cultivation of *S. cerevisiae* in a glucose-limited chemostat at a low D of 0.1 h^−1^. These mutations led to a progressive decrease of the residual glucose concentration over the course of 200 generations of evolution. Another example of increased substrate affinity of *S. cerevisiae* in glucose limited chemostats comes from Wu *et al.* [47]. They studied multiple steady states at different time points, and significant changes in the enzyme kinetics and metabolite concentrations over the course of evolution were found, accompanied by reductions in the residual glucose concentration. Tsen *et al.* [48] cultured *E. coli* in a lactose-limited chemostat for over 30 days at low dilution rates. They argued that because the control coefficient of the lactose permease on growth rate was relatively large, a small increase in the activity of this transporter should have a large impact on the growth rate and thus the fitness of the bacterium [49]. They indeed found a good correlation between the lactose permease activity and the growth rate of the evolved strains, which both increased during the course of evolution. They found that the K_M_ of the mutant permease was about half that of the wildtype and that the V_max_ had not changed.

Less attention has been given to increases in μ_max_ at high dilution rates in chemostats. However, several evolution experiments have been conducted at different dilution rates. In one of them, *Aspergillus niger* was evolved in glucose-limited chemostats at three different dilution rates, at Ds that correspond to 24%, 48% and 69% of the μ_max_. They found that the V_max_ of the glucose transporter increased about two-fold at increasing growth rates and that the apparent K_M_ of the transporter increased about 4.5-fold [50]. Similar behaviour was found in an evolution experiment of *E. coli* in chemostats with dilution rates of 0.1, 0.3 and 0.53 h^−1^. While the μ_max_ of the evolved strains compared to the wildtype increased significantly at increasing dilution rates, the Monod constant had decreased much less at the highest dilution rate [51]. This substantiates that the selection pressure on substrate affinity is relieved at increasing D and that the μ_max_ becomes more important.

Another change that can be observed at different dilution rates has to do with the metabolic pathways that are employed by an organism. A clear example of this comes from Postma *et al.* [43]. They found that *S. cerevisiae* uses the respiratory pathway at low dilution rates, while at higher dilution rates (*i.e.*, higher substrate concentrations) the metabolic flux shifts towards the fermentative pathway even under aerobic conditions, also referred to as the Crabtree effect (see Figure 6a). This shift in metabolic mode was not abrupt, a gradual change from respiration to fermentation was observed. The same observation was done by van Hoek *et al.* [52].

A theoretical perspective on the adaptations occurring in chemostats can be found in Beardmore *et al.* [53] (similar to the study by Gudelj *et al.* [54]). They report that it is possible for multiple genotypes to coexist in a chemostat, even though it is a constant environment. Using a stochastic mutation/selection model of evolution in the chemostat, they show how maintenance of multiple genotypes depends on the mutation rate and the existence of trade-offs (e.g., yield/rate; rate/affinity). At low mutation rates, the “fittest” is selected, while at high mutation rates the “flattest” is selected. The flattest has a small fitness difference upon a mutation, so a robust genotype, and a lower fitness peak. When several trade-offs occur between model parameters, a broader range of mutation rates exists for the maintenance of diversity. Such trade-offs result in a negative frequency dependence: an increase in frequency of high rate phenotypes depletes resources, leading to lower concentrations and thus higher selection for “flatter”, efficient cells, which in turn resets resource levels. These results are in line with chemostat experiments with yeast and *E. coli* that show a trade-off between high yield and high rate [44,55,56,57].

### 3.2. Fitness Effects of Changing Environments

In contrast to laboratory conditions, natural environments are often much more dynamic. Dynamics can, for example, occur in the type or amount of nutrients present; in the temperature; or in the presence of stresses. In this section, we discuss several examples of microbes that adjust to changing environments, and of the strategies they employ to increase fitness in these fluctuating environments.

The first example we will consider shows the importance of genetic adaptations in gene regulatory mechanisms in changing environments. Poelwijk *et al.* [58] constructed a strain with the *catR-SACB* gene cassette under the control of the lacI repressor to investigate evolution of regulatory systems in changing environments. Induction by IPTG in the presence of sucrose was detrimental (as the *SACB* gene converts it into a toxic product), while in the presence of chloramphenicol (CAM) it was beneficial (as the *catR* gene confers antibiotic resistance). When these two conditions were alternated, using different concentrations of CAM and sucrose, a negative trade-off was found between fitness in the two environments. A predictive model of fitness was made, based on the expression levels of the two genes. A mutant library was made of *lacI* (using error prone PCR approx. 10^6^ variants were made), and the resulting strains were less able to repress expression of the cassette. These mutants were subjected to a competition assay in which a switch from sucrose without IPTG to CAM with IPTG was made. In this case, the wildtype is in the optimal fitness state, while the mutants before selection had severely reduced fitness. After a growth competition experiment under these conditions, the mutants acquired wildtype levels of fitness. In addition, the mutants were subjected to a competition assay in which a switch from sucrose with IPTG to CAM without IPTG was made. In this instance, the wildtype strain was less fit than the mutant strains. After three rounds of mutations and selection the mutant strains showed increased fitness and reached the optimal state. Genetic analysis revealed that a single mutation was able to restore *lacI* to the original fitness state (induction by IPTG), while several mutations were needed before the function of *lacI* was inverted (molecular mechanism: lacI only binds the operator in presence of IPTG). This example shows that evolution finds the optimal solution to an artificially imposed trade-off when selection pressures shift under new environmental circumstances.

While the previous experiment was conducted in two well defined laboratory conditions, and mutations were directed to one specific gene, Saxer *et al.* [59] investigated the effect of subjecting two strains, derived from the human gut, to a complex rich medium and allowing the accumulation of spontaneous mutations over the course of 500 generations. They found that selected mutations did not affect a single cellular process, but that most mutations occurred in two global regulators, *rpoS* and *arcA*. Sigma factor 38, *rpoS*, was often found to acquire loss of function mutations or to be downregulated. *rpoS* is a protein that induces transcription of a number of stress proteins upon starvation when entering stationary phase, and other environmental stress conditions (see Section 3.3). *arcA* is also a global regulator with pleiotropic effects, it is part of a two-component signal transduction system that downregulates the expression of respiratory metabolic enzymes (involved in e.g., TCA cycle; amino acid metabolism; and electron transport chain). Mutations in *arcA* decreased its expression levels or even led to complete gene knock-outs, thereby upregulating the genes under its control. Upon mutations in these two genes, the starvation response was greatly reduced and nutrient uptake and utilization were enhanced. Both effects are in line with expectations with regard to the rich medium the microbes were subjected to. These finding are in agreement with many other studies that show global regulators to be a primary target for evolution when selection pressures shift due to changes in the environment [60,61,62,63,64,65].

Mutation and selection only allow for a slow adaptation response to a dynamic environment. A much quicker response can be attained by changing gene expression levels via regulatory mechanisms. In response to an environmental change, cells often need to quickly upregulate genes that were previously not needed. A metabolic rewiring for instance, could then be required for resumption of reproduction and an increase in fitness upon a shift in conditions. The research done by Zaslaver *et al.* [66] gives an example of this phenomenon. The activities of promoters associated with enzymes involved in amino acid biosynthesis (arginine, serine, methionine) were followed over time with GFP and luciferase constructs, in medium lacking the corresponding amino acid. The response times corresponded to the order of the pathway: enzymes that occur earlier in the biosynthesis pathway were brought to expression faster after subjection to medium lacking the corresponding amino acid. In addition, enzymes that were expressed earlier were more highly expressed. This was explained using a model where an enzyme-cost function was minimized, showing that high expression of the earlier enzymes leads to a quick response (boost of initial substrates) at lower costs, as it reduces the costs of enzyme dilution due to cell growth.

These works all focussed on a single shift in environmental circumstances. In the wild, microbes are often faced with temporal fluctuations. Preparation for a new environment can be costly. Proteins that are not needed in one environment might be required for survival or growth in the next, but the production of such unneeded proteins can reduce fitness (see Section 4.3). On the other hand, when a cell is suddenly faced with a new environment to which it is not adapted, it might be quickly outcompeted by cells that were “pre-adapted”. One can imagine a trade-off to exist between specializing for a certain environment (reducing costs of currently unneeded proteins, thereby maximizing fitness) and generalizing (being prepared for changes in the environment, and quickly resuming growth upon an environmental switch). How do populations of cells then deal with fluctuations of the environment?

An example that displays the difference between specialist and generalist strategies comes from New *et al.* [67]. They compared 18 different strains of *S. cerevisiae* growing on glucose only or on a mixture of glucose and a non-preferred carbon source. In the latter case, “catabolite repression” mechanisms ensure that the preferred carbon source is used first, while the metabolic pathways involved in the catabolism of the non-preferred carbon sources are repressed. When the preferred carbon source is nearly depleted, cells display a short adaptation period, during which catabolic genes for other carbon sources are upregulated. Accordingly, a period of reduced growth is observed during such transitions, referred to as the lag phase. Using both population level and single cell level growth measurements, they observed that the length of the lag phase differed greatly between the different strains. A longer lag phase was shown to have benefits. Cells that were shifted from a preferred to a non-preferred substrate and allowed to adapt to that, showed reduced growth rates upon a shift back to the preferred carbon source. This was in contrast with cells that did not yet adapt to the non-preferred substrate. Also, the expression of non-preferred substrate genes appeared to be costly as it reduced the growth rate on the preferred carbon source. On the other hand, shorter lag phases allowed for faster, sometimes even immediate, resumption of growth upon an environmental switch. As some strains had short lag phases, while others had long lag phases, it appears that evolutionary adaptation can lead to either a generalist or specialist strategy. Indeed, after evolution in a switching environment, two different strategies appeared: (i) strains with shorter lag phases and increased fitness upon environmental shifts but reduced growth rates on the preferred carbon source; or (ii) strains with increased growth rates on the preferred carbon source but longer lag phases upon a shift to a new carbon source. Genetic analysis showed that genes involved in catabolite repression were responsible for the changes in lag time duration. Longer lag times occurred when repression was tighter, and shorter lag times were associated with “leaky” expression. A mathematical model was constructed that predicted the evolutionary success of either a specialist or generalist strain, based on strain frequency and duration of exposure to either the preferred or non-preferred substrate. These model predictions were confirmed in competition experiments. Thus, whether a specialist or a generalist strategy is most beneficial depends on the nature of environmental fluctuations.

**Figure 7:**
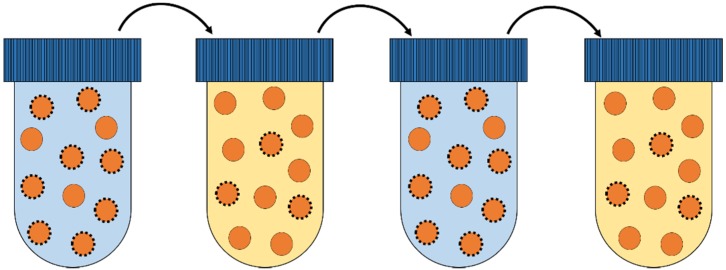
Cells with the same genotype can adapt to changing environments (different colour of tube content), by random switches between phenotypic states (dashed or non-dashed edges of cells). Phenotypic heterogeneity of a population of cells can allow quick resumption of growth upon a switch in the environment, as a subpopulation will be “preadapted” to the new circumstances.

As the maintenance of an environmental sensing system—like the catabolite repression machinery in the last example—entails fitness costs, randomly switching between phenotypic states can also be a beneficial strategy (see Figure 7). This was illustrated by Acar *et al.* [68]. They showed that fitness is enhanced when a fraction of the population is pre-adapted or “blindly anticipating” by means of stochastic switching between phenotypes in isogenic populations. They used an engineered *gal* pathway that allows experimental tuning of the rate of stochastic on/off switching of the uracil biosynthesis gene *Ura3*. In the absence of uracil, the “on” state is favoured (as uracil needs to be produced by the cells), while in an environment with uracil and 5FOA (a compound that is converted into a toxic product by Ura3) the “off” state is favoured. In a turbidostat culture, the environment was then changed between these two conditions. Fast switching of the cells between the on and off state allowed for quick recovery upon a change in the environment (as more cells are in the preferred state already), while slow switching allowed for a higher steady state growth rate (less cells switch back to the non-preferred state). They confirmed that this difference was due to the switching frequency of the cells, using a mathematical model. This model was subsequently used to predict that fast switching of environments favours fast switching cells, and slow switching of environments favours slow switching cells. Next, those predictions were experimentally confirmed.

Two theoretical papers that provide more proof that stochastic switching of cells can be optimized to fluctuating environments come from Gaál *et al.* [69] and Liberman *et al.* [70]. Both provide exact analytical results that depict how long term fitness can be enhanced by means of stochastic switching, and how it is influenced by the duration of each of the conditions, the impact on the fitness of phenotypes [69], whether the environment fluctuates randomly, and the mutation rate of the organism [70].

In summary, environmental dynamics shape evolution and force microbes to adapt to these fluctuations. Adaptations include genetic alterations (*i.e.*, mutations) and non-genetic changes (either regulated or stochastic). Together they allow microbes to grow in a multitude of conditions.

### 3.3. Environmental Stress Conditions

Some environments, instead of supporting growth, limit the growth rate associated fitness of an organism. When microbes are subjected to a stressful environment, the ability to survive extreme conditions becomes a vital aspect of their metabolic responses. Many conditions can be seen as microbial stressors, for instance: changes in pH, osmolarity, or temperature; nutrient starvation; pathogens; UV irradiation; oxidative stress; and antibiotic stress [71,72,73,74]. Cellular responses to these different types of stress often show similarities. In this section, we will discuss a number of adaptations that enable microbes to cope with unfavourable conditions.

The best understood cellular response to stressful conditions is the induction of the alternative *E. coli* sigma factor σ^S^, encoded by the *RpoS* gene (reviewed in [74]). When growth conditions vary, different sigma factors are selectively expressed to target RNA polymerase to specific sets of genes. The sigma factor σ^70^ is associated with growth supporting conditions, whereas σ^S^ is a stress response sigma factor, linked to nutrient starvation conditions (e.g., at the onset of the stationary phase) and various other stresses. σ^S^ is a “master” regulator associated with a myriad of physiological adaptations to unfavourable environments. It regulates up to 500 genes, of which approx. 140 belong to its “core regulon”, *i.e.*, genes that are always upregulated in response to increased σ^S^ levels. Amongst these genes are specific stress proteins, involved in, for example, coping with acidity; high osmolarity; or oxidative stress; but also proteins involved in the suppression of metabolism and induction of biofilm formation; the production of molecular chaperones; and membrane transporters (to dispose of harmful substances) [74]. The use of such a master regulator therefore enables the cell to respond to a large number of stresses at once, without the need for specific recognition machinery for each stress condition separately.

The evolution of antibiotic resistance, another stress, has been under extensive investigation in the last decades. Especially in light of the emergence of multi-resistant pathogens, it is vital to gain better understanding of the evolutionary trajectories microbes employ to guard themselves against modern medicine. The study of Weinrich *et al.* [24] has already been discussed in detail in Section 2, another good example comes from Toprak *et al.* [75]. In their research, genetic alterations leading to increasing antibiotic resistance to chloramphenicol (CAM), doxycycline (DOX) and trimetroprim (TMP) were followed during the course of evolution in a “morbidostat”. In this morbidostat, bacterial growth was monitored over time (ΔOD) and either fresh medium or medium supplemented with antibiotics was added to keep the population at half maximal growth rate (D = 0.5 μ_max_). When a mutation occurred that led to increased resistance, the concentration of antibiotic was increased. For CAM and DOX, the range of possible mutations (*i.e.*, mutational space) to increase resistance was large and quite some variation in the acquired mutations was found, leading to smooth phenotypic changes in the population. The affected genes included transcriptional, translational, and membrane proteins. Cross-resistance was observed for these two antibiotics; a mutation that confers resistance to DOX also led to CAM resistance and *vice versa*. This can be explained by the fact that both antibiotics target protein synthesis. For TMP, the mutational space proved more limited and was mostly directed at the folic acid synthesis gene DHFR, which is the direct target of TMP. This led to a stepwise change in phenotype, and hardly any cross-resistance was observed. The same mutations were found in previous (clinical) studies [76,77,78], and the order in which they appeared were very similar for the replicate populations. These studies show that, depending on the selective pressure of the stressor (e.g., target of the antibiotic), the possible mutational pathways may either be limited or broad.

The cross-stress protection observed in the last paper was also investigated by Dragosits *et al.* [73]. They evolved *E. coli* for 500 generations in environments containing four different stressors (high salinity, butanol, low pH and oxidative stress). For all four stresses, the fitness, determined by competition assays between evolved cells and their ancestor, increased after prolonged cultivation in the unfavourable environments. They found both positive cross-stress protection (e.g., strains evolved under butanol stress had increased fitness in the osmotic stress conditions and *vice versa*) and negative cross-stress reactions (e.g., a trade-off between butanol and oxidative stress seemed to emerge). A surprising result was that strains evolved under acidic stress conditions showed a lower fitness in acidic environments than the strains evolved under butanol or osmotic stress. This shows the existence of “evolutionary traps” that might be circumvented when adaptation takes place in a different environment (see Section 2). DNA sequencing and transcriptional profiling revealed a number of adaptations specific to the stress under which the strains were evolved, however some adaptations were shared among the positive cross-stress related strains. The butanol and osmotic stress evolved strains for example, showed mutations in the *fepA* gene involved in iron transport, which in previous research has been linked to toxic tolerance through changes in lipopolysaccharide biosynthesis [79,80].

In contrast to the cross-stress protection described above, the adaptation of *L. lactis* to growth at high temperature resulted repeatedly in a mutation in *gdpP*, a c-di-AMP phosphodiesterase, which is predicted to act as a membrane bound stress signalling protein. The identified mutants showed heat resistance but were at the same time hypersensitive to osmotic stress. The precise reason for this phenotype is not clear. In the *gdpP* mutants, the authors did find altered gene expression of *BusA*, a glycin-betaine uptake system involved in osmotic stress [81].

In conclusion, adaptations to stressful conditions comprise a large variety of both short-term changes through regulatory responses and long-term changes through mutation and selection. These adaptations enable microbes to survive extreme conditions, and—especially in natural surroundings—represent a vital part of microbial fitness.

## 4. Physical, Biochemical, and Cellular Constraints on Evolutionary Optimisation of Metabolism

The selection pressure in batch is on the growth rate of a cell, which most likely has an upper maximal value. This maximum is caused by many factors, including the thermodynamic properties of biochemical reactions, the need to properly partition proteins over cellular processes and the existence of trade-offs that might occur upon optimizing specific cellular properties. In this last section, we will discuss some of the constraints on evolution imposed by these inherent limitations on cellular growth.

### 4.1. Thermodynamics of Biochemistry Limits Cellular Processes

Cellular processes are inherently limited by the fact that they consist of biochemical reactions that obey thermodynamic laws and physical principles. A cell can only grow when it converts substrates into usable energy and “building blocks” (catabolism) from which it produces macromolecular components (anabolism), such as RNA, DNA, and proteins. All these processes are subject to constraints. For example, the replication of DNA is limited by the rate at which the helicase can denature the DNA into single strands and the rate of the primase to incorporate nucleotides into the new, growing DNA strand [82]. Considering the resulting maximal replication rate and the genome size of *E. coli*—knowing that DNA replication takes place in two directions from the origin of replication (ORI) at the same time—a minimal reproduction rate of approx. 40 min can be calculated [83]. Surprisingly, the minimal reproduction rate of an *E. coli* cell has been shown to be about half of that [84]. This can be explained by the fact that multiple replication origins and replication forks exist per cell, leading to the production of several DNA strands at the same time [85]. An exponentially growing *E. coli* cell at optimal conditions indeed has several (partial) chromosomal copies per cell [85,86]. It would thus appear that the DNA replication rate is not limiting to the cellular growth rate.

The growth rate of a cell is also limited by the translational rate. Indeed, growth rate has been found to be strongly dependent on protein synthesis rate set by the rate and the concentration of ribosomes. The extremes of the ribosome concentration set bounds to the generation time of a cell, with only one ribosome leading to a division time of approx. two years, and cells containing only ribosomes having a generation time of approx. 8 min [87]. As ribosomes themselves contain proteins, their maximal concentration is limited by the amount of resources available for protein synthesis [4] (see also Section 4.3). Klumpp *et al.* [88] showed that the rate at which ribosomes operate may depend mainly on the diffusion rate of charged tRNAs that bind to the ribosomal complex that is synthesizing protein. This diffusion rate is severely reduced inside the highly crowded cytosol of cells, compared to the normally observed rate of Brownian motion in non-crowded liquids. This physical limit on translation rate may therefore form a substantial constraint on cellular fitness.

A paper focussing on the physics of cells is by Dill *et al.* [87]. As the majority of the functionality of cells is derived from its enzymatic reactions, they focus on the properties of the proteome (the full collection of proteins of an organism). They first show that the folding stability of the protein shows a simple positive linear dependency on the number of amino acids incorporated into a protein. This is remarkable, as denaturation of proteins (as a result of e.g., temperature increases; changes in pH; or salinity of the environment) hardly seems to depend on other protein properties, such as hydrophobicity; number of hydrogen bonds; secondary or tertiary structure. Thus, given the known (measured) size distribution of proteins in an organism such as *E. coli*, the stability of its proteome can be predicted at given temperatures. They show that for *E. coli* grown at 37 °C, the stability of the proteome is already marginal, an increase of only 4 °C would destabilize the proteome by 16%, and the temperature at which most cells die (50 °C) coincides with a “denaturation catastrophe”. This emphasises the importance of stabilizing mutations during the course of protein evolution (see Section 4.3). On the other hand, enzymatic reactions have activation barriers in terms of free energy, an acceleration of metabolic processes may thus be expected upon temperature increases. This leads to the prediction of an optimal growth temperature for cells, at which the temperature is high enough for reactions to take place at a sufficient rate, while the majority of proteins have not been denatured. Their predictions were well in line with measured growth rates over a range of temperatures for a large number of microbial species [87].

Another physical aspect of cellular processes described in this paper is the previously mentioned diffusion rate, which depends on the radius of the diffusing molecule and the viscosity (crowdedness by other molecules) of the surroundings. Only a few parameters are needed to estimate the time for one molecule to travel a certain distance to another molecule, through a cell of a specific size, in order for a reaction to take place. This again leads to the prediction of an optimum at which biochemical reaction rates are maximized, in this case of the size and crowdedness of the cell. The authors assume that the protein amount of a cell is constant, thus a larger cell will have a lower degree of molecular crowding due to dilution (a justified assumption based on e.g., [3]). Thus, when a cell is too large, diffusion would take too long (too large a distance between two molecules), whereas when a cell is too small, diffusion would also take too long (too much crowding of molecules forming “obstacles” for diffusing molecules). Their predicted protein density is indeed in line with densities found in cells [87].

The optimal cell size and shape have also been looked at from another perspective by Koch [89,90]. He studied the optimal ratio between a cell’s surface area and volume. Smaller cells have a larger surface to volume (S/V) ratio than bigger cells, which has implications for the cellular strategies that can be attained. The main feature of a small cell is its ability to rapidly take up resources from the environment, as it has a large membrane area over which diffusion can take place, and in addition it can harbour a relatively large number of membrane proteins. It also allows rapid distribution of molecules throughout the cell. This would in theory allow a cell of small size to grow more rapidly than a bigger cell. Of course, there is a limit to being small: all cellular components necessary for growth in various environments (in terms of genetic material, ribosomes and metabolic protein) should be able to fit inside it. Large cells on the other hand are more limited in the rate at which substances can be taken up and distributed as a result of Fick’s diffusion law [91], and therefore often require additional machinery for transportation (e.g., endoplasmic reticulum) and subcellular compartmentalization of molecules with certain functions (organelles). Nonetheless, cell sizes ranging from 0.02–400 μm^3^ have been found to exist, displaying the remarkable versatility of sizes that support a high enough evolutionary fitness. The shape of a cell, however, has a much smaller influence on the fitness of a cell. A comparison between a rod shaped cell and a spherical cell showed that similar changes in S/V upon growth and cell division would occur leading to comparable growth rates at various points during the growth cycle [89,90].

Although there are obvious reasons to believe that physical constraints limit growth rates of microbial cells, it is intriguing that certain organisms successfully pushed these limits to the extreme. The fastest doubling time reported in literature is for *Chlostridium perfringens* at only 6.3 min [92]. Other reports of fast generation times include the organisms *Vibrio natriegens* (9.8 min [93] and 7 min [94]) and Thermobrachium celere (10 min [95]). As a general tendency, fast generation times seem to be reported mainly in thermophilic organisms [96], which is consistent with increased reaction and diffusion rates at high temperatures. These examples show that, given the right conditions, the generation times can be extremely short and that the Pareto fronts (limits) of the physical constrains might not be reached by many organisms.

### 4.2. Relation between Enzyme Benefits, Enzyme Costs and Cellular Fitness

**Figure 8:**
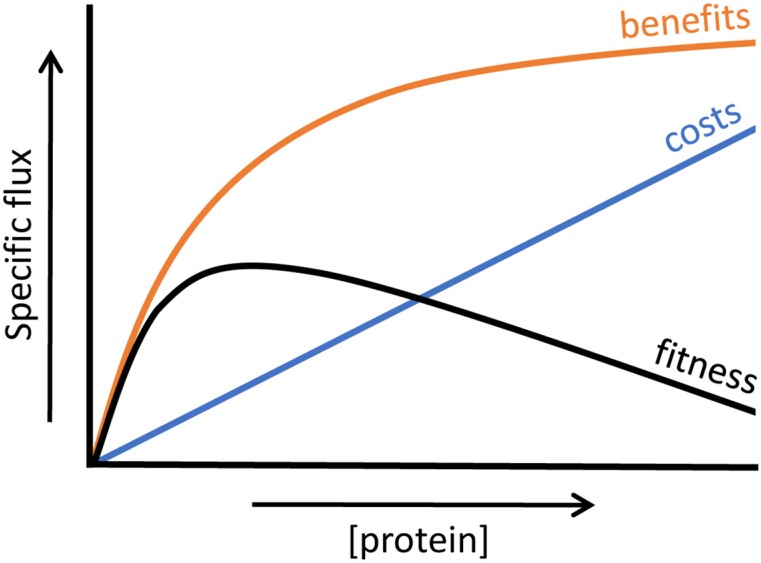
The costs (in blue) at increasing protein expression levels increase linearly, while benefits (in orange) will go towards a maximum. This leads to a fitness curve (in black) which shows an optimum at intermediate expression levels. Adapted from Berkhout *et al.* [97].

The fitness of a growing organism depends on its metabolic activity. Enzymes are responsible for taking up the available resources from the environment; catabolism to harvest energy and building blocks; cell wall synthesis; DNA replication; and so on. Hence, the expression of a protein can convey a direct contribution to cell growth, and thereby provide a fitness benefit. These benefits are thought to increase with protein expression levels (e.g., more enzyme increases catabolic flux), up to a certain maximum [97]. This maximum can, for example, be reached when in a pathway the enzyme at the initial rate-limiting step is expressed to such high levels that another step in the pathway becomes limiting. The shape of the benefit function at increasing protein concentrations can thus be represented as a saturating dependency (see Figure 8). In experimental terms, net benefits can be measured as the increase in growth rate upon higher induction of a gene that contributes to fitness.

On the other hand, protein expression also comes at a cost, in terms of the consumption of a cell’s resources. Many experiments have been conducted in which proteins that are not useful for a cell (*i.e.*, gratuitous proteins) are upregulated, leading to a decrease in growth rate. Dong *et al.* [98] found a substantial growth inhibition of growth rate-maximized cells when gratuitous proteins (truncated EF-Tu and β-galactosidase) were overexpressed. They tested an expression range of 0%–30% of the total protein content, and found a decrease in growth rates, with total growth inhibition at 30%. The simple protein burden model thus did not seem to be applicable, and they concluded this was caused by the accumulation of stress proteins and a reduction in ribosome concentrations. Another example comes from Stoebel *et al.* [99]. In a glucose limited chemostat, without lactose, they induced the *lac* operon to different levels using IPTG and competed three different mutants that lacked either *lacY* (the lactose permease responsible for lactose uptake), *lacZ* (β-galactosidase responsible for breaking lactose up into glucose and galactose) or *lacA* (an acetyl transferase of which the exact function is currently not fully understood). They found that 76.7% of the total cost is attributable to *lacZ* expression, the other 23.3% to *lacY* and *lacA*. As many factors can account for these costs of protein expression (e.g., nucleotide or amino acid incorporation; occupation of RNA polymerase, tRNA or ribosome; protein activities), they wondered what the main contribution to protein costs is. The costs were found to be proportional to gene size and protein concentrations. In addition, they tagged the lacZ protein with a sequence that targets it for protein degradation (thereby removing the cost contribution from enzyme activity) and found no effect on the cost of expression. They thus concluded that costs of protein expression are in the processes of RNA and protein synthesis. Elaborating more on this, Vind *et al.* [100] found that when the *lac* operon gets highly induced using IPTG in the absence of lactose, it reduces the translation of other proteins due to competition for ribosomes. The synthesis rate of other proteins (like *dnaK*, *rpsA*, *tuf*, and *tsf*) decreased after induction of the *lac* operon, and when lacZ entailed 25% of the total protein content, a 5% reduction in elongation rate of these other proteins was found. This is in line with the findings of Scott *et al.* [4]. They reported that the growth rate (tuned by nutrient quality) and ribosomal content (fraction RNA/ribosomal protein of total protein) show a positive linear correlation. When more ribosomes were inhibited by the antibiotic chloramphenicol, more ribosomes were synthesised and the growth rate decreased. They constructed a model that predicted a growth rate decrease upon unneeded protein synthesis because less protein was available for growth. These model predictions were then confirmed by experiments in which β-galactosidase was induced to different levels with IPTG.

In another experimental evolution study, an *L. lactis* strain, isolated from plant material, was cultured in milk [101]. This led to the loss of a 51 kb transposon that coded for genes involved in the utilization of typical plant oligosaccharides, such as melibiose, raffinose or stachyose. Since *L. lactis* is commonly found on plant material or in dairy environments, it is believed that the dairy strains evolved from plant isolates. The transposon disappeared almost completely from two independent populations in a period of about 150 generations, indicating the advantage of this genome erosion in the rich medium milk. At the same time, these strains lost their ability to grow on plant oligosaccharides.

Since protein expression comes at significant costs, fitness optimization cannot be simply achieved by increasing protein production. Fitness maximisation requires that the difference between the benefits and costs of a protein should be maximised, as was shown by Dekel & Alon [5]. They first measured the costs and benefits of the *lac* operon, by growing *E. coli* either in the absence of lactose, induced by IPTG (leading to a decreased growth rate) or at increasing concentrations of lactose (leading to increasing growth rates). Using a model, they predicted optimal *lac* activity levels, from benefit and cost consideration. The predicted optimal expression levels were indeed reached after 300–500 generations of evolution. In the absence of lactose, *lac* activity was completely lost and at several concentrations of lactose (ranging from 0.1–5 mM) the predicted optimal expression levels were reached.

The usefulness of benefit minus cost maximisation is further substantiated by the theoretical paper by Berkhout *et al.* [97]. They argued that metabolic enzyme concentrations must be optimized in such a way that the protein is not expressed at too high or too low concentrations. Since total protein content (resource) is constrained, costs were defined as the linear reduction in flux (fitness) as more of the unneeded enzyme is expressed and other enzymes redistributed according to the new reduced level of resources. Note that as costs depend on enzyme concentrations, a higher cost will be associated with proteins that have a short lifetime (*i.e.*, high degradation rate). The synthesis rate of these proteins needs to be higher (to avoid a decreasing concentration due to degradation) than for proteins that have long lifetimes. Enzyme benefits depend on metabolic system kinetics. Maximisation of benefit minus cost is depicted in Figure 8: when the benefits minus costs function (*i.e.*, fitness) shows a maximum, the highest metabolic flux can be attained. They also showed that the flux control coefficient of enzymes is related to their selection coefficient, which is often used in evolutionary studies, providing a link between biochemistry and microbial evolution.

A different cost–benefit relationship exists for extracellular proteins. Examples of such proteins are: yeast’s invertase that cleaves sucrose before it can be imported into the cell [102]; extracellular proteases that cleave proteins into peptides that can subsequently be taken up [103]; and siderophores that facilitate iron uptake [104]. Since cells that do not make extracellular proteins can still benefit from them, “cheater” cells can evolve that do not have the synthesis costs, only the benefits. Such systems lead to population dynamics where spatial separation and diffusion rates between cells determine the evolution and co-existence of various phenotypes [102,103].

### 4.3. Trade-Offs in Metabolic Evolution

**Figure 9:**
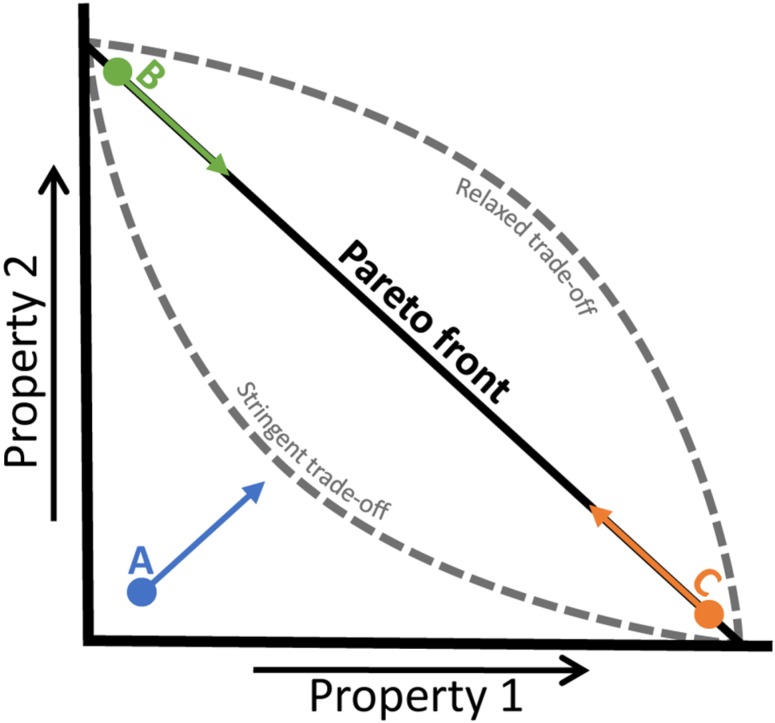
A trade-off between two cellular or molecular properties can be visualised as an apparent Pareto front (black line). When neither of the properties is maximized (point A), increases in both can be achieved (blue arrow). When for example property 2 has been fully optimized (point B), optimization of property 1 will require a reduction in property 2 (green arrow) and *vice versa* (orange arrow from point C). The more stringent the trade-off is, the more concave the Pareto front will become (lower dashed line), while if the trade-off is more relaxed, it can become convex (upper dashed line).

Many trade-offs have been reported to exist that each pose limitations to the optimization of microbial traits [8,15,16]. Trade-offs rule out that all traits can be optimized at the same time. In the case of a trade-off, the optimisation of one cellular property occurs at the expense of another. A trade-off can often be visualised as an apparent “Pareto front” that cannot be surpassed (Figure 9). A cell encountering a new environment to which it has not been optimized might show increases in both properties (point A in Figure 9), while a cell that has maximized one property will only be able to improve the other property at the expense of the former (points B and C in Figure 9). The shape of this Pareto front can vary and expresses the stringency of the trade-off: if the trade-off is very strong, the function might become concave (lower dashed line in Figure 9), while under more relaxed conditions it can become convex (upper dashed line in Figure 9). In this section, we will include some examples of trade-offs that have been encountered in evolution experiments, indicating limits to microbial adaptation.

The specialist *vs.* generalist trade off was already encountered in Section 3.2. Microbial strains can be optimized for fast growth on one specific carbon source, which goes at the expense of rapid adaptation to other carbon sources and *vice versa* [67]. A similar trade-off is found at the molecular level, in the form of a trade-off between the thermostability of a protein and its functionality [8]. Fasan *et al.* [105] found that the evolution of a cytochrome P450 fatty acid hydroxylase into a propane monooxygenase initially led to a great broadening of the enzyme substrate range at the expense of protein stability. During a subsequent increase in rate and efficiency of the enzyme on propane this range again narrowed. Another example comes from Wang *et al.* [106]. They showed that isolated protein from mutants of antibiotic β-lactamase TEM-1 with increased cephalosporin resistance, had decreased thermostability and reduced catalytic rates for their former target, penicillins. This stability loss arose from a reshaping of the active site and could be restored by a second mutation distant from the active site. It thus appeared that due to epistatic effects, mutations that led to newly acquired functions of proteins were associated with a decrease in stability, and should be accompanied by other mutations to avoid that function is compromised by reduced stability.

Sometimes, evolution can lead to the discovery of unexpected trade-offs that are very species specific. Meyer *et al.* [107], for example, evolved *E. coli* for over 45,000 generations in a medium where the sole carbon source was glucose. The resulting mutants, in contrast to their ancestors, were immune to λ-phage infections, without having encountered this virus during the course of evolution. It turned out that these phages rely on the maltose uptake system LamB to infect bacteria, and that the expression of this system had been severely reduced in the absence of maltose to reduce costs (see Section 4.3). A trade-off between the ability to metabolize certain substrates and the resistance to a certain virus thus became apparent.

The most well-known example of a trade-off might be between metabolic yield and rate. This trade-off has a theoretical basis in the first thermodynamic principles. For a chemical reaction, the energy difference (ΔG) between a substrate and product can partly be invested in the production of ATP, but if the reaction were to have a maximal ATP yield, no energy would be left over to drive the reaction forward; hence, to increase the rate, the yield must decrease [41,108,109]. The existence of this trade-off for isolated reactions occurring at or near thermodynamic equilibrium has hardly been disputed. However, in microbes metabolism consists of extensive networks of reactions, with only a small subset producing ATP, and the constraint of thermodynamic equilibrium might not always be satisfied. In addition, biomass yield is not the same as ATP yield. Experimental research to substantiate the existence of this yield/rate trade-off in living organisms is thus vital. In fact, an increasing number of papers report that this trade-off might indeed exist also at the cellular level. Jasmin *et al.* [33] and Weusthuis *et al.* [57] found that the biomass yield of *S. cerevisiae* decreased when prolonged chemostat cultivation led to evolved strains showing increased growth rates. Postma *et al.* [43] showed that increasing dilution rates in a chemostat (*i.e.*, increasing growth rates) were negatively correlated with the biomass yield, and explained this by the observed shift in metabolic pathways employed by the yeast strain (see Figure 5 and Figure 6). At low growth rates, the flux was directed mainly through the highly efficient respiratory pathway, while at higher growth rates the main fraction of carbon flux went through the high rate fermentative pathway. This was further substantiated by the *in silico* self-replicator model of Molenaar *et al.* [42] that showed how different metabolic strategies can optimize fitness based on the natural selection imposed by the environment. Apart from yeast, this same trend was found for other organisms. In the LTEE, Novak *et al.* [56] found a within-population negative correlation between rate and yield of evolved *E. coli* cells. Furthermore, in the aforementioned paper by Bachmann *et al.* [29], the trajectories over the yield/rate landscape of the high yield mutant and revertant high rate phenotypes were followed. The yield/rate trade-off appeared as a Pareto front. This, again, coincided with a shift from high yield, mixed acid fermentation to high rate, lactic acid fermentation.

In short, a large number of factors pose constraints on the optimization of fitness by microbial cells. Biophysics, as well as biochemistry, can explain these limits. Such limits cause fitness trade-offs and shape microbial evolution. They reduce phenotypic flexibility and force evolution.

## 5. Concluding Remarks

In this review, we discussed a large number of factors that influence microbial fitness. Spontaneous mutations together with stochastic or regulated phenotypic changes alter cellular properties like metabolic rate or yield, cell size, and stress resistance. This can be positively or negatively selected for in a certain environment, and allows organisms to adapt to a wide variety of conditions, be it stable or fluctuating, growth supporting or stressful. These adaptations are limited by physical constraints, trade-offs and the genetic background, leaving only a select number of possible strategies to optimize the phenotype and acquire a maximal fitness.

Over the last decades, the growing realization that experiments and theoretical work can augment each other has led to a systematic approach for expanding our detailed knowledge of the workings of evolution and the cellular features that arise from that. This knowledge has already been applied to our advantage, for example, in the fields of biotechnology and pharmaceuticals.

A whole cell perspective, for instance on a cell’s metabolism or protein economy, has led to the identification of regulatory mechanisms and trade-offs that emerge at such high levels of organisation, in addition to those occurring at the level of single proteins and genes. The nonlinear relation that exists between the phenotype and genotype of a cell is another important insight. This underlies, for instance, the phenomenon of diminishing returns and various types of epistatic interactions, together leading to preferred mutation trajectories in fitness landscapes. Also, this knowledge can be of use in biotechnological settings, and most certainly for understanding the rapid evolution of (multi-)antibiotics resistant strains. In addition, the realisation that the choice of the cultivation method can select for particular organisms with desired properties is an approach with great promises for biotechnological strain development. Finally, the interplay between environmental time scales and those of phenotypic and genetic adaptations will undoubtedly lead to many more fundamental insights into the limits and potential of microbial evolution.

In conclusion, the realization that selection pressure, organismal fitness, cellular strategy, biochemical implementation and biochemical and physical constraints should always be viewed in relation to one another, allows a deeper understanding of the processes underlying evolution and the resulting phenotypic properties of microorganisms.

## References

[1] Barrick J.E., Lenski R.E. (2013). Genome dynamics during experimental evolution. Nat. Rev. Genet..

[2] Klumpp S., Hwa T. (2014). Bacterial growth: Global effects on gene expression, growth feedback and proteome partition. Curr. Opin. Biotechnol..

[3] Klumpp S., Zhang Z., Hwa T. (2009). Growth rate-dependent global effects on gene expression in bacteria. Cell.

[4] Scott M., Gunderson C.W., Mateescu E.M., Zhang Z., Hwa T. (2010). Interdependence of cell growth and gene expression: Origins and consequences. Science.

[5] Dekel E., Alon U. (2005). Optimality and evolutionary tuning of the expression level of a protein. Nature.

[6] Berkhout J., Bruggeman F.J., Teusink B. (2012). Optimality Principles in the Regulation of Metabolic Networks. Metabolites.

[7] Wright S. (1932). The roles of mutation, inbreeding, crossbreeding and selection in evolution. Proc. Int. Congr. Genet..

[8] Romero P.A., Arnold F.H. (2009). Exploring protein fitness landscapes by directed evolution. Nat. Rev. Mol. Cell Biol..

[9] Smith J.M. (1970). Natural selection and the concept of a protein space. Nature.

[10] Näsvall J., Sun L., Roth J.R., Andersson D.I. (2012). Real-time evolution of new genes by innovation, amplification, and divergence. Science.

[11] Alberts B., Johnson A., Lewis J., Raff M., Roberts K., Walter P. (2008). Molecular Biology of the Cell.

[12] Ochman H., Lawrence J.G., Groisman E.A. (2000). Lateral gene transfer and the nature of bacterial innovation. Nature.

[13] Poelwijk F.J., Kiviet D.J., Weinreich D.M., Tans S.J. (2007). Empirical fitness landscapes reveal accessible evolutionary paths. Nature.

[14] Nagel A.C., Joyce P., Wichman H.A., Miller C.R. (2012). Stickbreaking: A novel fitness landscape model that harbors epistasis and is consistent with commonly observed patterns of adaptive evolution. Genetics.

[15] Elena S.F., Lenski R.E. (2003). Evolution experiments with microorganisms: The dynamics and genetic bases of adaptation. Nat. Rev. Genet..

[16] Kussell E. (2013). Evolution in microbes. Annu. Rev. Biophys..

[17] Chou H.-H., Chiu H.-C., Delaney N.F., Segrè D., Marx C.J. (2011). Diminishing returns epistasis among beneficial mutations decelerates adaptation. Science.

[18] De Vos M.G.J., Poelwijk F.J., Battich N., Ndika J.D.T., Tans S.J. (2013). Environmental dependence of genetic constraint. PLoS Genet..

[19] Koonin E.V., Wolf Y.I. (2010). Constraints and plasticity in genome and molecular-phenome evolution. Nat. Rev. Genet..

[20] Wagner A. (2008). Robustness and evolvability: A paradox resolved. Proc. Biol. Sci..

[21] Wielgoss S., Barrick J.E., Tenaillon O., Wiser M.J., Dittmar W.J., Cruveiller S., Chane-Woon-Ming B., Médigue C., Lenski R.E., Schneider D. (2013). Mutation rate dynamics in a bacterial population reflect tension between adaptation and genetic load. Proc. Natl. Acad. Sci. USA.

[22] Wiser M.J., Ribeck N., Lenski R.E. (2013). Long-term dynamics of adaptation in asexual populations. Science.

[23] McDonald M.J., Hsieh Y.-Y., Yu Y.-H., Chang S.-L., Leu J.-Y. (2012). The evolution of low mutation rates in experimental mutator populations of Saccharomyces cerevisiae. Curr. Biol..

[24] Weinreich D.M., Delaney N.F., Depristo M.A., Hartl D.L. (2006). Darwinian evolution can follow only very few mutational paths to fitter proteins. Science.

[25] Couñago R., Chen S., Shamoo Y. (2006). *In vivo* molecular evolution reveals biophysical origins of organismal fitness. Mol. Cell.

[26] Peña M.I., Davlieva M., Bennett M.R., Olson J.S., Shamoo Y. (2010). Evolutionary fates within a microbial population highlight an essential role for protein folding during natural selection. Mol. Syst. Biol..

[27] Lobkovsky A.E., Koonin E.V. (2012). Replaying the tape of life: Quantification of the predictability of evolution. Front. Genet..

[28] Carneiro M., Hartl D.L. (2010). Colloquium papers: Adaptive landscapes and protein evolution. Proc. Natl. Acad. Sci. USA.

[29] Bachmann H., Fischlechner M., Rabbers I., Barfa N., Branco dos Santos F., Molenaar D., Teusink B. (2013). Availability of public goods shapes the evolution of competing metabolic strategies. Proc. Natl. Acad. Sci. USA.

[30] Bryson V., Szybalski W. (1952). Microbial Selection. Science.

[31] Bull A.T. (2010). The renaissance of continuous culture in the post-genomics age. J. Ind. Microbiol. Biotechnol..

[32] Ibarra R.U., Edwards J.S., Palsson B.O. (2002). Escherichia coli K-12 undergoes adaptive evolution to achieve in silico predicted optimal growth. Nature.

[33] Jasmin J.-N., Dillon M.M., Zeyl C. (2012). The yield of experimental yeast populations declines during selection. Proc. Biol. Sci..

[34] Zeyl C., Vanderford T., Carter M. (2003). An evolutionary advantage of haploidy in large yeast populations. Science.

[35] Wortel M.T., Peters H., Hulshof J., Teusink B., Bruggeman F.J. (2014). Metabolic states with maximal specific rate carry flux through an elementary flux mode. FEBS J..

[36] Müller S., Regensburger G., Steuer R. (2014). Enzyme allocation problems in kinetic metabolic networks: Optimal solutions are elementary flux modes. J. Theor. Biol..

[37] Schuster S., Fell D.A., Dandekar T. (2000). A general definition of metabolic pathways useful for systematic organization and analysis of complex metabolic networks. Nat. Biotechnol..

[38] Dragosits M., Mattanovich D. (2013). Adaptive laboratory evolution—Principles and applications for biotechnology. Microb. Cell Factories.

[39] Schuster S., Pfeiffer T., Fell D.A. (2008). Is maximization of molar yield in metabolic networks favoured by evolution?. J. Theor. Biol..

[40] Teusink B., Wiersma A., Molenaar D., Francke C., de Vos W.M., Siezen R.J., Smid E.J. (2006). Analysis of growth of Lactobacillus plantarum WCFS1 on a complex medium using a genome-scale metabolic model. J. Biol. Chem..

[41] MacLean R.C. (2008). The tragedy of the commons in microbial populations: Insights from theoretical, comparative and experimental studies. Heredity (Edinb)..

[42] Molenaar D., van Berlo R., de Ridder D., Teusink B. (2009). Shifts in growth strategies reflect tradeoffs in cellular economics. Mol. Syst. Biol..

[43] Postma E., Verduyn C., Scheffers W.A., Van Dijken J.P. (1989). Enzymic analysis of the crabtree effect in glucose-limited chemostat cultures of Saccharomyces cerevisiae. Appl. Environ. Microbiol..

[44] Lipson D.A., Monson R.K., Schmidt S.K., Weintraub M.N. (2008). The trade-off between growth rate and yield in microbial communities and the consequences for under-snow soil respiration in a high elevation coniferous forest. Biogeochemistry.

[45] Pfeiffer T., Schuster S., Bonhoeffer S. (2001). Cooperation and competition in the evolution of ATP-producing pathways. Science.

[46] Jansen M.L.A., Diderich J.A., Mashego M., Hassane A., de Winde J.H., Daran-Lapujade P., Pronk J.T. (2005). Prolonged selection in aerobic, glucose-limited chemostat cultures of Saccharomyces cerevisiae causes a partial loss of glycolytic capacity. Microbiology.

[47] Wu L., Mashego M.R., Proell A.M., Vinke J.L., Ras C., van Dam J., van Winden W.A., van Gulik W.M., Heijnen J.J. (2006). *In vivo* kinetics of primary metabolism in Saccharomyces cerevisiae studied through prolonged chemostat cultivation. Metab. Eng..

[48] Tsen S.D., Lai S.C., Pang C.P., Lee J.I., Wilson T.H. (1996). Chemostat selection of an Escherichia coli mutant containing permease with enhanced lactose affinity. Biochem. Biophys. Res. Commun..

[49] Dykhuizen D.E., Dean A.M., Hartl D.L. (1987). Metabolic flux and fitness. Genetics.

[50] Jørgensen T.R., vanKuyk P.A., Poulsen B.R., Ruijter G.J.G., Visser J., Iversen J.J.L. (2007). Glucose uptake and growth of glucose-limited chemostat cultures of Aspergillus niger and a disruptant lacking MstA, a high-affinity glucose transporter. Microbiology.

[51] Wick L.M., Weilenmann H., Egli T. (2002). The apparent clock-like evolution of Escherichia coli in glucose-limited chemostats is reproducible at large but not at small population sizes and can be explained with Monod kinetics. Microbiology.

[52] Van Hoek P., Van Dijken J.P., Pronk J.T. (1998). Effect of specific growth rate on fermentative capacity of baker’s yeast. Appl. Environ. Microbiol..

[53] Beardmore R.E., Gudelj I., Lipson D.A., Hurst L.D. (2011). Metabolic trade-offs and the maintenance of the fittest and the flattest. Nature.

[54] Gudelj I., Beardmore R.E., Arkin S.S., MacLean R.C. (2007). Constraints on microbial metabolism drive evolutionary diversification in homogeneous environments. J. Evol. Biol..

[55] Maharjan R.P., Seeto S., Ferenci T. (2007). Divergence and redundancy of transport and metabolic rate-yield strategies in a single Escherichia coli population. J. Bacteriol..

[56] Novak M., Pfeiffer T., Lenski R.E., Sauer U., Bonhoeffer S. (2006). Experimental tests for an evolutionary trade-off between growth rate and yield in *E. coli*. Am. Nat..

[57] Weusthuis R.A., Pronk J.T., van den Broek P.J., van Dijken J.P. (1994). Chemostat cultivation as a tool for studies on sugar transport in yeasts. Microbiol. Rev..

[58] Poelwijk F.J., de Vos M.G.J., Tans S.J. (2011). Tradeoffs and optimality in the evolution of gene regulation. Cell.

[59] Saxer G., Krepps M.D., Merkley E.D., Ansong C., Deatherage Kaiser B.L., Valovska M.-T., Ristic N., Yeh P.T., Prakash V.P., Leiser O.P. (2014). Mutations in Global Regulators Lead to Metabolic Selection during Adaptation to Complex Environments. PLoS Genet..

[60] Notley-McRobb L., Seeto S., Ferenci T. (2003). The influence of cellular physiology on the initiation of mutational pathways in Escherichia coli populations. Proc. Biol. Sci..

[61] Blank D., Wolf L., Ackermann M., Silander O.K. (2014). The predictability of molecular evolution during functional innovation. Proc. Natl. Acad. Sci. USA.

[62] Maharjan R., Seeto S., Notley-McRobb L., Ferenci T. (2006). Clonal adaptive radiation in a constant environment. Science.

[63] Philippe N., Crozat E., Lenski R.E., Schneider D. (2007). Evolution of global regulatory networks during a long-term experiment with Escherichia coli. Bioessays.

[64] Shimizu K. (2013). Metabolic Regulation of a Bacterial Cell System with Emphasis on Escherichia coli Metabolism. ISRN Biochem..

[65] Puentes-Téllez P.E., Kovács Á.T., Kuipers O.P., van Elsas J.D. (2014). Comparative genomics and transcriptomics analysis of experimentally evolved Escherichia coli MC1000 in complex environments. Environ. Microbiol..

[66] Zaslaver A., Mayo A.E., Rosenberg R., Bashkin P., Sberro H., Tsalyuk M., Surette M.G., Alon U. (2004). Just-in-time transcription program in metabolic pathways. Nat. Genet..

[67] New A.M., Cerulus B., Govers S.K., Perez-Samper G., Zhu B., Boogmans S., Xavier J.B., Verstrepen K.J. (2014). Different levels of catabolite repression optimize growth in stable and variable environments. PLoS Biol..

[68] Acar M., Mettetal J.T., van Oudenaarden A. (2008). Stochastic switching as a survival strategy in fluctuating environments. Nat. Genet..

[69] Gaál B., Pitchford J.W., Wood A.J. (2010). Exact results for the evolution of stochastic switching in variable asymmetric environments. Genetics.

[70] Liberman U., Van Cleve J., Feldman M.W. (2011). On the evolution of mutation in changing environments: Recombination and phenotypic switching. Genetics.

[71] Poole K. (2012). Bacterial stress responses as determinants of antimicrobial resistance. J. Antimicrob. Chemother..

[72] Shimizu K. (2013). Regulation Systems of Bacteria such as Escherichia coli in Response to Nutrient Limitation and Environmental Stresses. Metabolites.

[73] Dragosits M., Mozhayskiy V., Quinones-Soto S., Park J., Tagkopoulos I. (2013). Evolutionary potential, cross-stress behavior and the genetic basis of acquired stress resistance in Escherichia coli. Mol. Syst. Biol..

[74] Landini P., Egli T., Wolf J., Lacour S. (2014). sigmaS, a major player in the response to environmental stresses in Escherichia coli: Role, regulation and mechanisms of promoter recognition. Environ. Microbiol. Rep..

[75] Toprak E., Veres A., Michel J.-B., Chait R., Hartl D.L., Kishony R. (2012). Evolutionary paths to antibiotic resistance under dynamically sustained drug selection. Nat. Genet..

[76] Huovinen P. (1987). Trimethoprim resistance. Antimicrob. Agents Chemother..

[77] Smith D.R., Calvo J.M. (1982). Nucleotide sequence of dihydrofolate reductase genes from trimethoprim-resistant mutants of Escherichia coli. Evidence that dihydrofolate reductase interacts with another essential gene product. Mol. Gen. Genet..

[78] Flensburg J., Sköld O. (1987). Massive overproduction of dihydrofolate reductase in bacteria as a response to the use of trimethoprim. Eur. J. Biochem..

[79] Hagiwara D., Yamashino T., Mizuno T. (2004). A Genome-wide view of the Escherichia coli BasS-BasR two-component system implicated in iron-responses. Biosci. Biotechnol. Biochem..

[80] Reyes L.H., Almario M.P., Winkler J., Orozco M.M., Kao K.C. (2012). Visualizing evolution in real time to determine the molecular mechanisms of n-butanol tolerance in Escherichia coli. Metab. Eng..

[81] Smith W.M., Pham T.H., Lei L., Dou J., Soomro A.H., Beatson S.A., Dykes G.A., Turner M.S. (2012). Heat resistance and salt hypersensitivity in Lactococcus lactis due to spontaneous mutation of llmg_1816 (gdpP) induced by high-temperature growth. Appl. Environ. Microbiol..

[82] Marians K.J. (2008). Understanding how the replisome works. Nat. Struct. Mol. Biol..

[83] Milo R., Jorgensen P., Moran U., Weber G., Springer M. (2010). BioNumbers—The database of key numbers in molecular and cell biology. Nucleic Acids Res..

[84] Reshes G., Vanounou S., Fishov I., Feingold M. (2008). Timing the start of division in *E. coli*: A single-cell study. Phys. Biol..

[85] Bremer H., Dennis P., Neidhardt F.C. (1996). Modulation of chemical composition and other parameters of the cell by growth rate. Escherichia coli and Salmonella: Cellular and Molecular Biology.

[86] Atkinson M.R., Savageau M.A., Myers J.T., Ninfa A.J. (2003). Development of Genetic Circuitry Exhibiting Toggle Switch or Oscillatory Behavior in Escherichia coli. Cell.

[87] Dill K.A., Ghosh K., Schmit J.D. (2011). Physical limits of cells and proteomes. Proc. Natl. Acad. Sci. USA.

[88] Klumpp S., Scott M., Pedersen S., Hwa T. (2013). Molecular crowding limits translation and cell growth. Proc. Natl. Acad. Sci. USA.

[89] Koch A. (2001). The Mechanical Aspects of Cell. Bacterial Growth and Form.

[90] Koch A.L. (1996). What size should a bacterium be? A question of scale. Annu. Rev. Microbiol..

[91] Fick A. (1995). On liquid diffusion. J. Membr. Sci..

[92] Labbe R.G., Huang T.H. (1995). Generation Times and Modeling of Enterotoxin-Positive and Enterotoxin-Negative Strains of Clostridium perfringens in Laboratory Media and Ground Beef. J. Food Prot..

[93] Eagon R.G. (1962). Pseudomonas natriegens, a marine bacterium with a generation time of less than 10 min. J. Bacteriol..

[94] Maida I., Bosi E., Perrin E., Papaleo M.C., Orlandini V., Fondi M., Fani R., Wiegel J., Bianconi G., Canganella F. (2013). Draft Genome Sequence of the Fast-Growing Bacterium Vibrio natriegens Strain DSMZ 759. Genome Announc..

[95] Engle M., Li Y., Rainey F., DeBlois S., Mai V., Reichert A., Mayer F., Messner P., Wiegel J. (1996). Thermobrachium celere gen. nov., sp. nov., a rapidly growing thermophilic, alkalitolerant, and proteolytic obligate anaerobe. Int. J. Syst. Bacteriol..

[96] Burgess S.A., Lindsay D., Flint S.H. (2010). Thermophilic bacilli and their importance in dairy processing. Int. J. Food Microbiol..

[97] Berkhout J., Bosdriesz E., Nikerel E., Molenaar D., de Ridder D., Teusink B., Bruggeman F.J. (2013). How biochemical constraints of cellular growth shape evolutionary adaptations in metabolism. Genetics.

[98] Dong H., Nilsson L., Kurland C.G. (1995). Gratuitous overexpression of genes in Escherichia coli leads to growth inhibition and ribosome destruction. J. Bacteriol..

[99] Stoebel D.M., Dean A.M., Dykhuizen D.E. (2008). The cost of expression of Escherichia coli lac operon proteins is in the process, not in the products. Genetics.

[100] Vind J., Sørensen M.A., Rasmussen M.D., Pedersen S. (1993). Synthesis of proteins in Escherichia coli is limited by the concentration of free ribosomes. Expression from reporter genes does not always reflect functional mRNA levels. J. Mol. Biol..

[101] Bachmann H., Starrenburg M.J.C., Molenaar D., Kleerebezem M., van Hylckama Vlieg J.E.T. (2012). Microbial domestication signatures of Lactococcus lactis can be reproduced by experimental evolution. Genome Res..

[102] Gore J., Youk H., van Oudenaarden A. (2009). Snowdrift game dynamics and facultative cheating in yeast. Nature.

[103] Bachmann H., Molenaar D., Kleerebezem M., van Hylckama Vlieg J.E.T. (2011). High local substrate availability stabilizes a cooperative trait. ISME J..

[104] Buckling A., Harrison F., Vos M., Brockhurst M.A., Gardner A., West S.A., Griffin A. (2007). Siderophore-mediated cooperation and virulence in Pseudomonas aeruginosa. FEMS Microbiol. Ecol..

[105] Fasan R., Meharenna Y.T., Snow C.D., Poulos T.L., Arnold F.H. (2008). Evolutionary history of a specialized p450 propane monooxygenase. J. Mol. Biol..

[106] Wang X., Minasov G., Shoichet B.K. (2002). Evolution of an antibiotic resistance enzyme constrained by stability and activity trade-offs. J. Mol. Biol..

[107] Meyer J.R., Agrawal A.A., Quick R.T., Dobias D.T., Schneider D., Lenski R.E. (2010). Parallel changes in host resistance to viral infection during 45,000 generations of relaxed selection. Evolution.

[108] Pfeiffer T., Bonhoeffer S. (2002). Evolutionary Consequences of Tradeoffs between Yield and Rate of ATP Production. Z. Phys. Chem..

[109] Waddell T.G., Repovic P., Meléndez-Hevia E., Heinrich R., Montero F. (1999). Optimization of glycolysis: New discussions. Biochem. Educ..

